# Design, Synthesis, Molecular Modeling and Antitumor Evaluation of Novel Indolyl-Pyrimidine Derivatives with EGFR Inhibitory Activity

**DOI:** 10.3390/molecules26071838

**Published:** 2021-03-25

**Authors:** Naglaa M. Ahmed, Mahmoud M. Youns, Moustafa K. Soltan, Ahmed M. Said

**Affiliations:** 1 Pharmaceutical Organic Chemistry Department, Faculty of Pharmacy, Helwan University, Ein-Helwan, Helwan, Cairo 11795, Egypt; nogamoon2005@yahoo.com; 2 Biochemistry Department, Faculty of Pharmacy, Helwan University, Ein-Helwan, Helwan, Cairo 11795, Egypt; dr.mahmoudyouns@yahoo.com; 3 Oman College of Health Sciences, Muscat 123, Oman; mostafakhames3@yahoo.com; 4 Medicinal Chemistry Department, Faculty of Pharmacy, Zagazig University, Zagazig 44519, Egypt; 5 Department of Chemistry, University at Buffalo, The State University of New York, Buffalo, NY 14260, USA

**Keywords:** cancer, pyrimidine, indole, drug design, EGFR, molecular modeling

## Abstract

Scaffolds hybridization is a well-known drug design strategy for antitumor agents. Herein, series of novel indolyl-pyrimidine hybrids were synthesized and evaluated in vitro and in vivo for their antitumor activity. The in vitro antiproliferative activity of all compounds was obtained against MCF-7, HepG2, and HCT-116 cancer cell lines, as well as against WI38 normal cells using the resazurin assay. Compounds **1**–**4** showed broad spectrum cytotoxic activity against all these cancer cell lines compared to normal cells. Compound **4g** showed potent antiproliferative activity against these cell lines (IC_50_ = 5.1, 5.02, and 6.6 μM, respectively) comparable to the standard treatment (5-FU and erlotinib). In addition, the most promising group of compounds was further evaluated for their in vivo antitumor efficacy against EAC tumor bearing mice. Notably, compound **4g** showed the most potent in vivo antitumor activity. The most active compounds were evaluated for their EGFR inhibitory (range 53–79%) activity. Compound **4g** was found to be the most active compound against EGFR (IC_50_ = 0.25 µM) showing equipotency as the reference treatment (erlotinib). Molecular modeling study was performed on compound **4g** revealed a proper binding of this compound inside the EGFR active site comparable to erlotinib. The data suggest that compound **4g** could be used as a potential anticancer agent.

## 1. Introduction

Cancer is an abnormal cell growth. It has the potential of migrating from organ to organ in the human body, this process is called metastasis [1,2,3]. The high mortality rate from cancer is due to its ability to metastasis and spread among multiple organs [4]. Cancer is generally formed by self-cell gene mutations or by manipulating normal cell differentiation [5]. The leading factors that could initiate cancer formation are drugs, viruses, radiation, smoking, or even diet [6]. Cancer is classified by the World Health Organization (WHO) as the second leading cause of death worldwide with 9.6 million deaths in 2018 [7]. The death rate from cancer is projected to continue rising worldwide, with an estimated 13 million deaths in 2030 [8]. In fact, cancer treatment is challenging because of the lack of specificity of the treatment on only malignant cells; normal cells are also affected. The current standard care for cancer patients involves surgery, radiation, immunotherapy, and chemotherapy [9]. Given all of that, there is a high demand for the discovery of new, safer, and efficient anticancer agents. 

**Figure 1:**
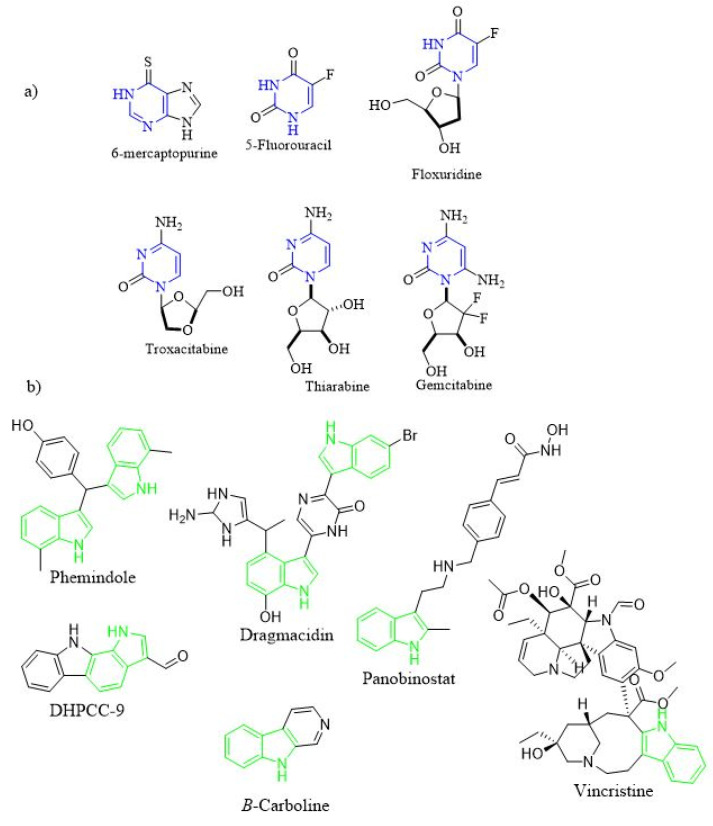
(**a**) Pyrimidine containing compounds as anticancer agents; (**b**) indole containing compounds with antitumor activity.

Heterocyclic scaffolds have attracted considerable attention in the design of many biologically active synthetic compounds [10,11,12,13,14]. Pyrimidine, six-membered ring heterocyclic ring with two nitrogen atoms, is widely present in several natural and synthetic drugs [15]. Thus, there are many synthetic pyrimidine derivatives with exhibited several pharmacological actions such as antimicrobial [16,17], anticancer [18,19,20], antioxidant [18], antifungal [21], antithyroid [22], antiviral [23,24,25], anticonvulsant [26], anti-inflammatory [27], antitubercular [28], antileishmanial [29], antimalarial [30], anthelminthic [31], anti-HIV [32], antitubercular [33], and antidepressant activities [34]. In addition, pyrimidine ring prevails in naturally formed structures such alkaloids [35], nucleic bases [36], vitamins [37], and numerous other pharmacophores. Pyrimidine ring is considered the core scaffold of the genetic material and hence it is crucial for anticancer activity [38]. In recent years, several research groups revealed many pyrimidine containing compounds that have anticancer activity (Figure 1a). The FDA approved pyrimidine derivatives with anticancer activity was classified into three primary classes based on their mechanism of action (MOA) [39]: (1) thiopurines: such as 6-mercaptopurine; (2) fluoropyrimidines: such as 5-Fluorouracil (5-FU) and Floxuridine; (3) deoxynucleoside analogues: such as Gemcitabine, Thiarabine and Troxacitabine. Moreover, other pyrimidine compounds with other MOA were discovered such as: (1) glycogen synthase kinase (GSK) inhibitors [40]; (2) cyclin-dependent kinase (CDK) inhibitors [41], and (3) epidermal growth factor receptor (EGFR) inhibitors [42]. 

Another important heterocyclic nucleus is the indole ring. It is found in many natural and synthetic compounds including anticancer agents [43] (Figure 1b). Such compounds were found to act as antitumor agents via different MOAs [44] i.e., antimitotic agents (i.e., Vincristine), histone deacetylases (HDACs) (i.e., Panobinostat), PIM kinases (i.e., DHPCC-9), and DNA topoisomerases (i.e., β-carboline). 

**Figure 2:**
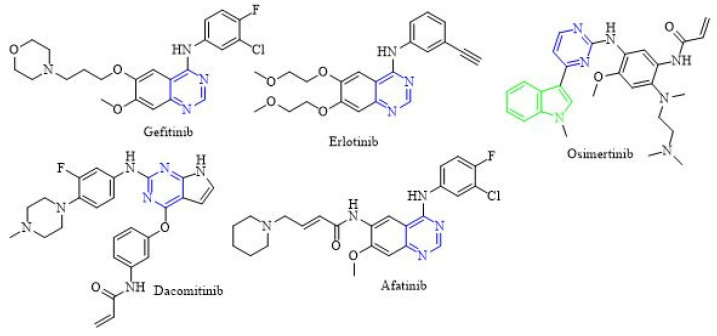
Examples of tyrosine kinase inhibitors that inhibit EGFR.

Scaffold hybridization is a drug design approach that is widely used in modern medicinal chemistry. It involves the combination of two or more known pharmacophores/scaffolds into the same structure. In this study, two major classes of anticancer scaffolds are being hybridized to form our novel compounds. The first class of anticancer scaffold is PAC-1 [45]. Dissecting PAC-1 structure, it consists of three regions (A-region, B-region, and C-regions). The B-region usually serves as a linker between A and C-regions. Previous studies on the structure-activity relationships of PAC-1 and its analogues have revealed that the acyl hydrazone moiety (B-region) plays a crucial role for their broad anticancer activity. The second class of anticancer compounds is EGFR inhibitors. Epidermal growth factor receptor (EGFR), also known as HER1 (human epidermal growth factor receptor 1), is an important subfamily of the protein kinases. EGFR is a key mediator that plays a crucial role in cellular functions [46]. The overexpression of EGFR was extensively studied because of its direct role in the development of many human solid tumors such as non-small cell lung cancer (NSCLC) [47] and breast cancer [48]. Therefore, it is expected that inhibition of EGFR protein kinase could be a potential therapeutic treatment for some prominent cancer types. There are two main classes of EGFR inhibitors [49]: (a) Small molecule tyrosine kinase inhibitors (TKIs): This class of compounds is three generations (Figure 2): (1) The first-generation EGFR inhibitors i.e., erlotinib and gefitinib; (2) The second-generation EGFR inhibitors i.e., afatinib and dacomitinib; (3) The third-generation EGFR inhibitors i.e., osimertinib; (b) Monoclonal antibodies (mAbs): There are two approved mAbs Cetuximab and Panitumumab. 

**Figure 3:**
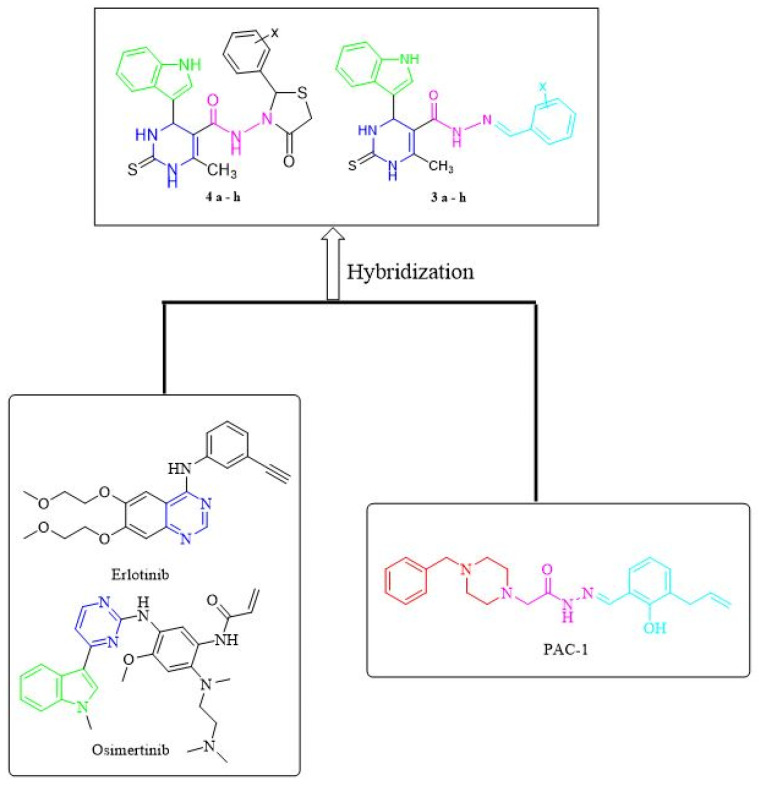
Rational design for the novel indolyl pyrimidine scaffold.

In this study, the design of our novel compounds was performed using a scaffold hybridization approach. These compounds are designed based on the combination of two pharmacophores (pyrimidine and indole) to form a hybrid scaffold (Figure 3). In addition, the acyl hydrazone moiety was used to bioisosterically replace the NH- of the EGFR inhibitor nucleus. All the synthesized indolyl pyrimidine hybrids were screened for their in vitro antiproliferative activity against three human cancer cell lines i.e., breast (MCF-7), liver (HepG2) and colon (HCT-116) as well as against lung fibroblast (WI38) normal cell line. This was followed-up by an in vivo antitumor evaluation using EAC tumor bearing mice for the most promising compounds. Moreover, these compounds were tested against EGFR enzymes to point out their anticancer mode of action. This complete study of structure–activity relationship (SAR) on these newly discovered compounds is expected to point out the route for more effective heterocyclic compounds as anticancer agents.

## 2. Results and Discussion

### 2.1. Chemistry 

**Scheme 1:**
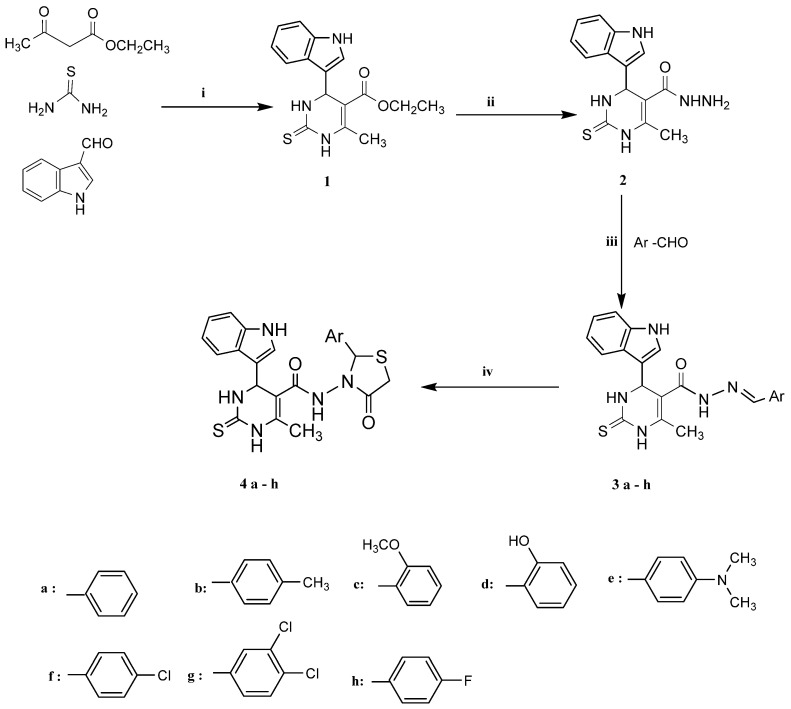
Synthesis of the designed compounds (**4a**–**h**). Reagents and conditions: (i) EtOH, HCl; (ii) NH_2_NH_2_, EtOH, reflux 10 h; (iii) AcOH, reflux 5–8 h; (iv) thioglycolic acid, AcOH, reflux 5–8 h.

Compound **1** was synthesized through one-pot three component Biginelli reaction [50,51] using an indole-3-carbaldehyde, ethyl acetoacetate, and thiourea. The mixture was stirred under reflux in ethanol containing few drops of 37% HCl. This was followed-up by hydrazinolysis of the ester group of intermediate **1** using hydrazine hydrate in ethanol to form compound **2**. Then, hydrazone derivatives **3a**–**h** were prepared from compound **2** via heating with the appropriate aromatic aldehydes either in absolute ethanol only or with acetic acid as a catalyst [52] (Scheme 1).

Furthermore, cyclo-condensation of compounds **3a**–**h** with thioglycolic acid in glacial acetic acid was performed to afford compounds **4a**–**h** (Scheme 1). The structure confirmation of these compounds was performed using MS, IR, ^1^H-NMR, ^13^C-NMR as well as elemental analysis. 

### 2.2. Molecular Modeling

**Figure 4:**
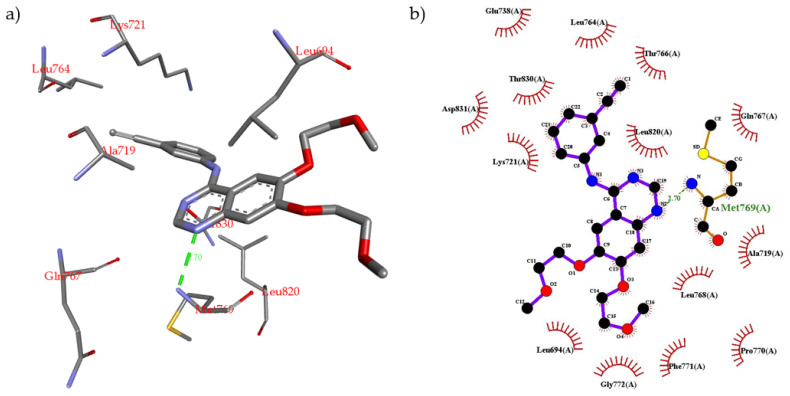

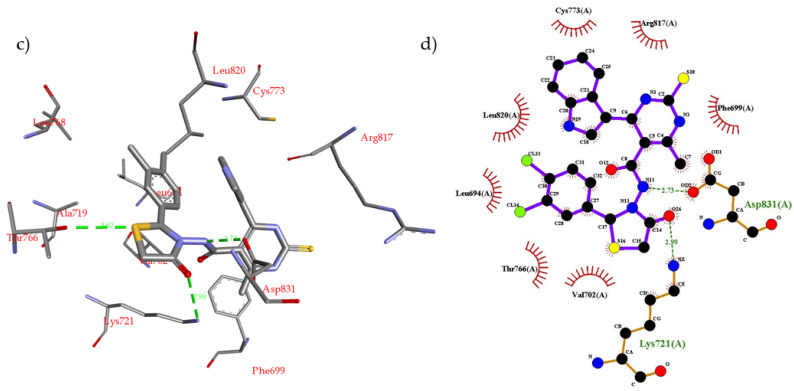
(**a**) Non-covalent interactions of erlotinib inside the EGFR active site; (**b**) 2D schematic representation of erlotinib inside the EGFR binding site; (**c**) non-covalent interactions of docked compound **4g** inside the EGFR active site; (**d**) 2D schematic representation of compound **4g** inside the EGFR binding site.

One of the cancer treatment approaches is to block the over activation of receptor tyrosine kinases (TKIs) signaling pathway i.e., inhibition of EGFR. The example for EGFR inhibitors is erlotinib, 1st generation TKIs. To investigate the protein–ligand interactions between EGFR and the novel scaffold, and the possible binding modes were predicted by molecular docking using Autodock Vina version 1.1.2 [53]. The binding mode of the most promising compound **4g** was compared to that of erlotinib in complex with the EGFR binding site. The crystal structure of EGFR kinase domain in complex with erlotinib was obtained from the protein data bank (PDB ID: 1M17) [54]. The docking parameters were validated by docking erlotinib into the EGFR binding site, and the binding mode was compared to the X-ray crystal structure. The docking results of erlotinib show nine possible conformers with binding energy ranging from −6.8 kcal/mol to −7.3 kcal/mol. The most stable (lowest energy) conformer was compared to the X-ray crystal structure. This conformer closely reproduced the binding mode observed crystallographically in PDB entry 1M17 forming the same interactions with EGFR active site (see Supplementary Materials). Analysis of X-ray crystal structure of erlotinib inbound to EGFR reveals that erlotinib (N-1 of quinazoline) interacts with the backbone NH of amino acid Met769 via an H-bond (2.70 Å). In addition, erlotinib phenyl rings form several hydrophobic interactions with proximal hydrophobic residues i.e., Val702, Ala719, Lys721, Leu764, Thr766, Leu820, and Thr830 (Figure 4a,b). Compound **4g** was docked inside the EGFR binding site using the same parameters used for erlotinib. It was found that compound **4g** binds more favorably than erlotinib inside the EGFR active site. The docking results of compound **4g** show nine possible conformers with binding energy ranging from −8.8 kcal/mol to −9.6 kcal/mol. Compound **4g** fits well inside the EGFR binding site and forms several non-covalent interactions (Figure 4c,d). The most stable conformer was used to interpret these non-covalent interactions. Compound **4g** was found to form three H-bonding interactions as follows: (1) The -NH of the linker forms an H-bond (2.73 Å) with Asp831 residue; (2) The -C=O of the thiazolidine ring forms an H-bond (2.99 Å) with Lys721 residue; (3) The S-atom of the thiazolidine ring forms weak H-bond (3.62 Å) with Thr766 residue. In addition, several strong hydrophobic interactions between the aromatic rings of compound **4g** and the proximal amino acid hydrophobic residues, i.e., Leu694, Phe699, Val702, Cys737, Thr766, and Leu820, were observed. 

### 2.3. Biological Screening 

#### 2.3.1. In Vitro Antiproliferative Activity

**Figure 5:**
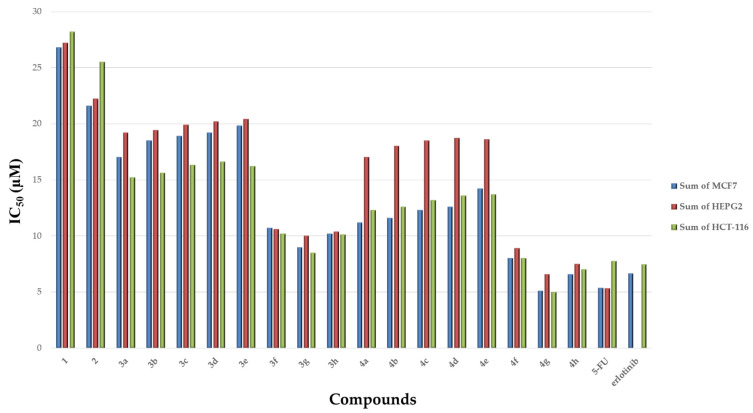
Cytotoxic activity (IC_50_) of compounds **1**–**4**, 5-FU and erlotinib in different cell lines.

Compounds **1**–**4** were assessed for their in vitro cytotoxic activity against three human cell lines i.e., breast (MCF-7), liver (HepG2), colon (HCT-116) cancer cell lines as well as in lung fibroblast (WI38) normal cell line using the Resazurin Cell Growth Inhibition Assay [55]. Both erlotinib and 5-Fluorouracil (5-FU) were used as reference drugs. Concentrations of the tested compounds that give about 50% inhibition of cell viability (IC_50_, mean of triplicate) in µM were obtained. The results revealed that most of the tested compounds showed significant in vitro antiproliferative activity when compared to normal cells. Compounds **1**–**2** were found to possess moderate antiproliferative activity against the three cell lines when compared to normal cell lines. Compound **4g** showed the most potent inhibitory profile against MCF-7, HepG2, and HCT-116 when compared to normal cell lines. In addition, compounds **4f** and **4h** showed promising inhibitory activity against all cell lines, whereas compounds **3f**, **3g**, and **3h** showed notable effectiveness against these cell lines. The IC_50_ range of the tested compounds was 8.30 to 20.4 μM. To interpret the in vitro results and correlate it to compounds’ structures (structure activity relation (SAR)), it was found that the presence of -COOEt (compound **1**) or -CONHNH_2_ (compound **2**) substituents on C5 of the pyrimidine nucleus resulted in moderate efficacy against MCF-7, HepG2, and HCT-116 cell line. However, if replaced by a hydrazone substituent, a more prominent efficacy toward the three cancer cell lines was observed. Notably, the inhibitory activity of compounds 3a-h is influenced by the type of the hydrazone formed. The most promising in vitro antiproliferative activity was obtained when the hydrazone is bearing electron-withdrawal groups i.e., 4-chlorobenzylidene moiety (compound **3f**), 3,4-dichloro benzylidene moiety (compound **3g**) and 4-fluorobenzylidene moiety (compound **3h**) compared to the standard treatment (Table 1, Figure 5). The inhibitory activity of compounds was reduced when the hydrazone group is bearing electron donating groups i.e., 4-methyl group (compound **3b**), 2-methoxy group (compound **3c**), 4-hydroxy (compound **3d**) and 4-dimethylamino group (compound **3e**). The trend of antiproliferative activity of compounds **3a**–**e** against all tested cell lines has the following descending order: (**3g** > **3h** >**3f** >**3a** >**3b** > **3c** > **3d** > **3e**). Furthermore, the addition of the thiazolidinone ring via a condensation reaction showed a more profound effectiveness against the three cancer cell lines compared to the standard treatment. The most potent compounds were found to follow what was revealed before that presence of an electron withdrawing group, i.e., 4-chlorophenyl (compound **4f**), 3,4-dichloro (compound **4g**) and 4-fluorophenyl (compound **4h**), improves the antiproliferative activity. The higher the lipophilic character of the electron withdrawing groups i.e., Cl on the phenyl ring, the more potent is the antiproliferative activity. In addition, installing an electron donating group was found to have no effect on the activity of these compounds (Table 1, Figure 4). All of the tested compounds **4a**–**h** showed in vitro antiproliferative activities with IC_50_ values ranging from 5.02 to 18.62 μM. The trend of antiproliferative activity of these hybrids **4a**–**e** against all tested cancer cell lines had the following descending order: (**4g** > **4h** > **4f** > **4a** > **4b** > **4c** > **4d** > **4e**). Compound **4g** showed the most potent activity against MCF-7, HepG2 and HCT-116 with IC_50_ = 5.1 ± 1.14, 5.02 ± 1.19 and 6.6 ± 1.40 μM, respectively, compared to the reference treatment (Table 1). This is mainly because this compound has the highest lipophilicity as well as less electron density as it has 2-Cl atoms (more electronegativity) on the indolyl pyrimidine-thiazolidinone.

**Figure 6:**
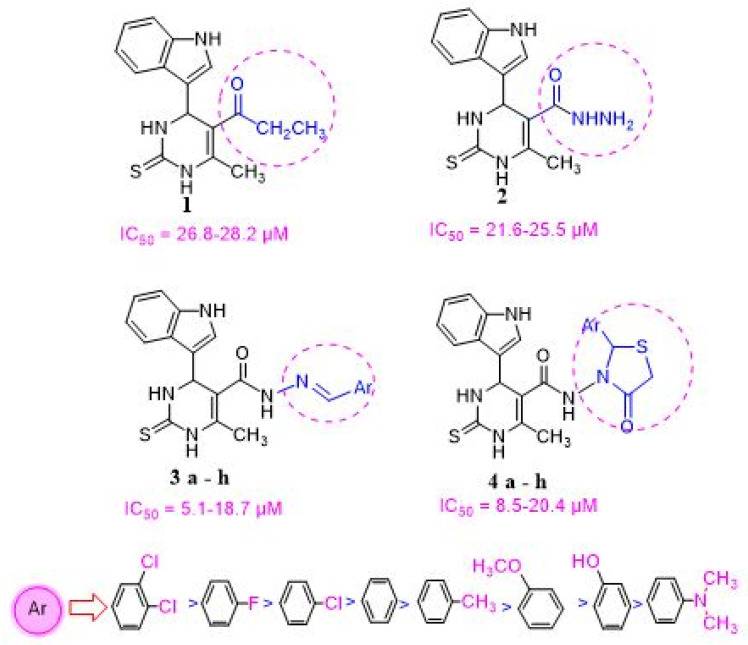
Structure activity relationship (SAR) of the pyrimidine derivatives against different cell lines.

As shown in Figure 6, there is a consistent relation between the lipophilicity and/or electronic property of the substituent groups on the Ar-group and the antiproliferative activity. It was found that the nature of substitution on the Ar group affected the order of activity of tested compounds against tested cell lines: (3,4-(Cl)_2_ > F > Cl > H > CH_3_ > OCH_3_ > OH > N(CH_3_)_2_). Installment of a more electronegative and a lipophilic substituent to phenyl ring on either the pyrimidine and thiazolidinone respectively enhance cytotoxic activity against MCF-7, HepG2, and HCT-116 cancer cells. In addition, the sensitivity of the tested cell lines to compounds **1**–**4** was found to be in the following descending order: (MCF-7, HCT-116 > HepG2). This is mainly because these cancer cell lines have different expression levels for EGFR. 

#### 2.3.2. In Vivo Antitumor Evaluation 

The most promising compounds with the best in vitro antitumor profile i.e., compounds **3g**, **4f**, **4g** and **4h** were further evaluated for their in vivo antitumor efficacy against Ehrlich ascites carcinoma (EAC) in mice. The antitumor activity of these compounds was assessed by the change in tumor volume, viable cell count, mean survival time (MST) and % increase in lifespan (% ILS) of the EAC tumor bearing mice and 5-FU (standard treatment) [56]. The mean survival time (MST) and % increase in lifespan (% ILS) of EAC tumor bearing mice, as well as the influence of these compounds on viable tumor cells and the tumor volume, are shown in Table 2. The hematological profile of these mice was also assessed in comparison with the standard treatment with 5-FU, and no obvious effect on RBCs or WBCs was observed. It was found that compound **4g** exhibited the highest % ILS of mice, as well as results illustrated that treatment with compound 4g resulted in the lowest tumor volume and viable tumor cell count. Tumor volume and viable tumor cell count were also determined, and results suggest that treatment with compound **4g** resulted in the lowest tumor volume and viable tumor cell count.

MST = (day of first death + day of last death)/2

%ILS = [(MST of treated group/MST of control group) − 1] × 100

#### 2.3.3. In Vitro EGFR Inhibition Assay

The correlation between the cytotoxic activity of compounds and the level of EGFR expression in the cancerous cell lines used was the cornerstone for an in vitro testing of the new hybrid compounds against EGFR. The data revealed that the sensitivity of the tested compounds to cell lines MCF-7, HCT-116 was significantly more than to the HepG2 cell lines. This is mainly because these cancerous cell lines exhibit different EGFR expression levels. The HepG2 cell line was found to have undetectable EGFR expression [57]. On the other hand, both MCF-7 and HCT-116 cell lines were found to have considerable EGFR expression [58,59]. WI38 cell line does not express EGFR. A first-generation EGFR inhibitor, erlotinib, was used as standard treatment. EGFR was treated with 10 µM of the chosen compounds as well as the reference treatment with erlotinib. The in vitro screening of these analogs against EGFR revealed inhibitory activity range 53–79%, and the best inhibitory activity 79% was shown by compound **4g** followed by **4f** (71%). The % inhibition of EGFR activity was measured for compounds **3g** (53%), and for compound **4h** (70%) (Table 3). IC_50_ for these compounds was determined. Most of the tested analogs showed comparable inhibitory activities to erlotinib (0.30 μM) as shown in Table 3. The tested analogs showed EGFR inhibitory activity with IC_50_ values ranging from 0.25 to 0.50 µM. Compound **4g** was found to be the most active one against EGFR comparable to erlotinib. In addition, compounds **4f** and **4h** exhibited good activity with IC_50_ value of 0.38 and 0.39 µM, respectively.

## 3. Materials and Methods

### 3.1. Instruments

All melting points were determined using Electro-thermal IA 9100 apparatus (Shimadzu, Kyoto, Japan) and were uncorrected. FT-IR spectra were recorded using potassium bromide pellets on a PerkinElmer 1650 spectrophotometer (USA). ^1^H-NMR and ^13^C-NMR spectra were obtained in DMSO-*d*_6_ using a Varian Mercury (300 MHz and 75 MHz respectively) spectrometer (Varian, Crawley, UK), and chemical shifts were given as ppm from TMS as internal reference. Mass spectra were recorded on 70 eV EI Ms-QP 1000 EX. Microanalyses were performed using Vario, Elementar apparatus, Organic Microanalysis Unit, Faculty of Science, Cairo University, Cairo, Egypt, and the results were within the accepted range (0.40) of the calculated values. Column Chromatography was performed on (Merck, Darmstadt, Germany) Silica gel 60 (particle size 0.06–0.20 mm).

### 3.2. Chemistry

#### 3.2.1. Ethyl 4-(1*H*-indol-3-yl)-6-methyl-2-thioxo-1,2,3,4-tetrahydropyrimidine-5-Carboxylate (**1**)

A mixture of thiourea (10 mmol), indole-3-carbaldehyde (10 mmol), and ethyl acetoacetate (10 mmol) in absolute ethanol (25 mL) containing 37% HCl (7 drops) was heated to reflux for 7 h, the reaction mixture was allowed to cool down to room temperature and was neutralized with 25% ammonia (0.5 mL). The precipitate formed was filtered off, washed several times with 50% ethanol, and was finally recrystallized from ethanol to give compound **1**, m.p. 245–247 °C as reported [50]. 

#### 3.2.2. 4-(1*H*-indol-3-yl)-6-methyl-2-thioxo-1,2,3,4-tetrahydropyrimidine-5-carbohydrazide (**2**)

A mixture of the ester derivative **1** (10 mmol) and hydrazine hydrate (50 mmol, 99%) in 20 mL ethanol was refluxed for 10 h, then was cooled down to room temperature and poured into ice/water mixture. The produced precipitate was filtered off, dried, and was crystallized from ethanol to give compound **2**, m.p. 208–210 °C as reported [50]. 

#### 3.2.3. General Procedure for the Preparation of Compounds (3**a**–**h**)

A mixture of compound **2** (10 mmol) and the appropriate aldehyde (20 mmol) in glacial acetic acid (35 mL) was refluxed for 5–8 h. The solution was cooled down to room temperature and ice was added, the formed precipitate was filtered off, washed with water, and was further crystallized from ethanol to give the desired hydrazone (3**a**–**h**). The titled compounds **3a**, **3d**, **3f**, and **3h** were synthesized according to the reported method [50].

##### 4-(1*H*-indol-3-yl)-6-methyl-2-thioxo-1,2,3,4-tetrahydropyrimidine-5-carboxylic acid (4-methyl-benzylidene)-hydrazide (**3b**)

Yellow crystals, yield 80%, m.p. 240–242 °C. IR (KBr) v max (cm^−1^): 3340 (NH), 1685 (C=O), 1590 (C=N). ^1^H-NMR (300 MHz, DMSO-*d*_6_) 2.1 (s, 3H, CH_3_), 2.5 (s, 3H, CH_3_, pyrim), 5.2 (s, 1H, pyrimidine), 6.6–8.2 (m, 9H, Ar–H), 8.7 (s, 1H, CH=N), 10.4,10.7,10.9, 11.5 (4 s, 4H, 4NH, D_2_O exchangeable). ^13^C-NMR (300 MHz, DMSO-*d*_6_) 18.0 and 20.0 (2CH_3_), 55.4 (C-4 pyrimidine), 106.5 (C-5 pyrimidine), 111.2–140.2 (aromatic C), 151.1 (C-6 pyrimidine), 156.0 (C=N), 168.1 (C=O), 178.2 (C=S); MS(EI): *m*/*z* 403 [M^+^] (14%); Anal. Calcd. for C_22_H_21_N_5_OS (403.5): C, 65.49; H, 5.25; N, 17.36; Found: C, 65.40; H, 5.20; N, 17.31.

##### 4-(1*H*-indol-3-yl)-6-methyl-2-thioxo-1,2,3,4-tetrahydropyrimidine-5-carboxylic acid (2-methoy- benzylidene)-hydrazide (**3c**)

Yellow crystals, yield 82%, m.p. 220–222 °C. IR (KBr) v max (cm^−1^): 3366 (NH), 1689 (C=O), 1604 (C=N). ^1^H-NMR (300 MHz, DMSO-*d*_6_) 2.5 (s, 3H, CH_3_, pyrim), 3.3 (s, 3H, OCH_3_), 5.0 (s, 1H, pyrimidine), 7.0–8.7 (m, 9H, Ar–H), 8.8 (s, 1H, CH=N), 9.6,10.0,10.4,11.6 (4s, 4H, 4NH, D_2_Oexchangeable). ^13^C-NMR (300 MHz, DMSO-*d*_6_) 18.3 (CH_3_), 58.2 (OCH_3_), 55.3 (C-4 pyrimidine), 106.8 (C-5 pyrimidine), 118.6–149.0 (aromatic C), 150.7 (C-6 pyrimidine), 155.2 (C=N), 165.2 (C=O), 177.6 (C=S). MS (EI): *m*/*z* 419 [M^+^] (11%). Anal. Calcd. for C_22_H_21_N_5_O_2_S (419.49): C, 62.99; H, 5.05; N, 16.69; Found: C, 62.90; H, 5.00; N, 16.61.

##### 4-(1*H*-indol-3-yl)-6-methyl-2-thioxo-1,2,3,4-tetrahydropyrimidine-5-carboxylic acid (4-dimethylamino-benzylidene)-hydrazide (**3e**)

Yellowish brown solid, yield 76%, m.p. 180–182 °C. IR (KBr) v max (cm^−1^): 3442 (NH),1685 (C=O), 1603 (C=N). ^1^H-NMR (300 MHz, DMSO-*d*_6_) 2.0 (s, 3H, CH_3_, pyrim), 2.5 (s, 6H, CH3), 5.0 (s, 1H, pyrimidine), 6.7–8.2 (m, 9H, Ar–H), 8.7(s, 1H, CH=N), 9.6,10.0,10.4, 11.0 (4s, 4H, 4NH, D_2_Oexchangeable). ^13^C-NMR (300 MHz, DMSO-*d*_6_) 18.0 (CH_3_), 43.6 (N-CH3), 55.0 (C-4 pyrimidine), 111.6-137.0 (aromatic Cs), 108.0 (C-5 pyrimidine), 153.0 (C-6 pyrimidine), 158.0 (C=N), 168.8 (C=O), 178.2 (C=S). MS(EI): *m*/*z* 432 [M^+^] (15 %). Anal. Calcd. for C_23_H_24_N_6_OS (432.17): C, 63.87; H, 5.59; N, 19.43; Found: 63.80; H, 5.51; N, 19.42.

##### 4-(1*H*-indol-3-yl)-6-methyl-2-thioxo-1,2,3,4-tetrahydropyrimidine-5-carboxylic acid (3,4-dichloro- benzylidene)-hydrazide (**3g**)

Brown crystals, yield 64%, m.p. 160–162 °C. IR (KBr) v max (cm^−1^): 3350 (NH), 1685 (C=O), 1686 (C=N). ^1^H-NMR (300 MHz, DMSO-*d*_6_) 2.50 (s, 3H, CH_3,_ pyrim), 5.2 (s, 1H, pyrimidine), 7.2–8.2 (m, 8H, Ar-H), 8.7 (s, 1H, CH=N), 9.9, 10.0,10.1,11.1 (4s, 4H, 4NH, D_2_O exchangeable). ^13^C-NMR (300 MHz, DMSO-*d*_6_) 18.2 (CH_3_), 55.3 (C-4 pyrimidine), 106.6 (C-5 pyrimidine), 111.0–140.4 (aromatic Cs), 151.3 (C-6 pyrimidine),154.2 (C=N), 178.4 (C=S), 186.6 (C=O). MS (EI): *m*/*z* 457 [M^+^] (12%), 459 [M^+2^] (4%). Anal. Calcd. for C_21_H_17_Cl_2_N_5_OS (458.3636): C, 55.03; H, 3.74; N, 15.28; Found: C, 55.05; H, 3.77; N, 15.20.

#### 3.2.4. General Procedure for the Preparation of Compounds (**4a**–**h**)

A mixture of compounds **3a**–**h** (10 mmol) and thioglycolic acid (10 mmol) in glacial acetic acid (30 mL) was refluxed for 5–8 h. The excess solvent was evaporated under vacuum, and the obtained residue was poured on crushed ice. The formed solid product was filtered off, washed with water, and was crystallized from ethanol to obtain the desired products **4a**–**h**, respectively.

##### 4-(1*H*-indol-3-yl)-6-methyl-*N*-(4-oxo-2-phenylthiazolidin-3-yl)-2-thioxo-1,2,3,4-tetrahydropyrimidine-5-carboxamide (**4a**)

Yellow solid, yield 80%, m.p. 270–272 °C. IR (KBr) v max (cm^−1^): 3390 (NH), 1654,1704 (2 C=O), 3060 (CH-Ar); ^1^H-NMR (300 MHz, DMSO-*d*_6_) 2.5 (s, 3H, CH_3_), 3.8 (s, 2H, CH_2_ of thiazolidinone ring), 5.9 (1H, s, CH of thiazolidinone ring), 5.1 (s, 1H, pyrimidine), 7.4–8.7 (m, 10H, Ar-H), 9.8,10.1,10.8, 11.0 (4s, 4H, 4NH, D_2_O exchangeable). ^13^C-NMR (300 MHz, DMSO-*d*_6_) 18.9 (CH_3_), 58.2 (CH), 39.3 (CH_2_), 55.5 (C-4 pyrimidine), 113.9–148.4 (aromatic Cs), 106.0 (C-5 pyrimidine), 155.3 (C-6 pyrimidine), 165.6 and 169.6 (2 C=O), 179.4 (C=S); MS (EI): *m*/*z* 463 [M^+^] (5%). Anal. Calcd. for C_23_H_21_N_5_O_2_S_2_ (463.57): C, 59.59; H, 4.57; N, 15.11; Found: C, 59.55; H, 4.55; N, 15.10.

##### 4-(1*H*-indol-3-yl)-6-methyl-*N*-(4-oxo-2-(p-tolyl)thiazolidin-3-yl)-2-thioxo-1,2,3,4-tetrahydropyrimidine-5-carboxamide (**4b**)

Off-white solid, yield 80%, m.p. 220–222 °C. IR (KBr) v max (cm^−1^): 3260 (NH), 1665 and 1690 (2 C=O), 3050 (CH-Ar), 2910 (CH-sp3), ^1^H-NMR (300 MHz, DMSO-*d*_6_) 2.5–2.6 (s, 6H, 2CH_3_), 3.90 (2H, s, CH_2_ of thiazolidinone ring), 5.90 (1H, s, CH of thiazolidinone ring), 5.1 (s, 1H,pyrimidine), 6.7–8.5 (m, 10H, Ar-H), 10.0, 10.2, 10.6, 11.0 (4 s, 4H, 4NH, D_2_O exchangeable); ^13^C-NMR (300 MHz, DMSO-*d*_6_) 18.1 and 20.1 (2 CH_3_), 57.0 (CH), 38.0 (CH_2_), 55.2 (C-4 pyrimidine), 111.0–140.1 (aromatic Cs), 106.1 (C-5 pyrimidine), 152.1 (C-6 pyrimidine), 165.0 and 168.1 (2 C=O), 179.0 (C=S); MS(EI): *m*/*z* 477 [M^+^] (8%). Anal. Calcd. for C_24_H_23_N_5_O_2_S_2_ (477.60): C, 60.36; H, 4.85; N, 14.66; Found: C, 60.34; H, 4.82; N, 14.60.

##### 4-(1*H*-indol-3-yl)-*N*-(2-(2-methoxyphenyl)-4-oxothiazolidin-3-yl)-6-methyl-2-thioxo-1,2,3,4-tetrahydropyrimidine-5-carboxamide (**4c**)

Buff solid, yield 80%, m.p. 200–202 °C. IR (KBr) v max (cm^−1^): 3255 (NH), 1660 and 1693 (2 C=O), 3090 (CH-Ar), 2940 (CH-sp^3^). ^1^H-NMR (300 MHz, DMSO-*d*_6_) 2.3 (s, 3H, CH_3_), 3.3 (s, 3H, OCH_3_), 4.0 (2H, s, CH_2_ of thiazolidinone ring), 5.9 (1H, s, CH of thiazolidinone ring), 5.1 (s, 1H, pyrimidine), 7.2–7.3 (m, 9H, Ar-H), 9.6, 10.3, 10.8, 11.2 (4s, 4H, 4NH, D_2_O exchangeable); ^13^C-NMR (300 MHz, DMSO-*d*_6_) 18.5 (CH_3_), 59.9 (OCH_3_), 58.5 (CH), 39.3 (CH_2_), 55.2 (C-4 pyrimidine), 113.5-145.3 (aromatic Cs), 108.1 (C-5 pyrimidine), 155.5 (C-6 pyrimidine), 166.3 and 168.4 (2 C=O), 179.3 (C=S). MS(EI): *m*/*z* 493 [M^+^] (10%). Anal. Calcd. for C_24_H_23_N_5_O_3_S_2_ (493.12): C, 58.40; H, 4.70; N, 14.19; Found: C, 58.45; H, 4.77; N, 14.15.

##### *N*-(2-(2-hydroxyphenyl)-4-oxothiazolidin-3-yl)-4-(1*H*-indol-3-yl)-6-methyl-2-thioxo-1,2,3,4-tetrahydropyrimidine-5-carboxamide (**4d**)

Yellow crystals, yield 75%, m.p.180–182 °C. IR (KBr) v max (cm^−1^): 3250(NH), 1662 and 1692 (2 C=O), 3070 (CH-Ar), 2930 (CH-sp^3^); ^1^H-NMR (300 MHz, DMSO-*d*_6_) 2.6 (s, 3H, CH_3_), 3.92 (2H, s, CH_2_ of thiazolidinone ring), 5.92 (1H, s, CH of thiazolidinone ring), 5.0 (s, 1H, pyrimidine), 6.5-8.6 (m, 9H, Ar-H), 11.2 (s, 1H, OH, D_2_Oexchangeable), 10.0, 10.2, 10.8, 11.0 (s, 4H, 4NH, D_2_O exchangeable); ^13^C-NMR (300 MHz, DMSO-*d*_6_) 18.2 (CH_3_), 57.0 (CH), 38.0 (CH_2_), 55.0 (C-4 pyrimidine), 112.0–140.0 (aromatic Cs), 106.2 (C-5 pyrimidine), 152.0 (C-6 pyrimidine), 165.2 and 168.3 (2 C=O), 179.2 (C=S). MS (EI): *m*/*z* 479 [M^+^] (11%). Anal. Calcd. for C_23_H_21_N_5_O_3_S_2_ (479.57): C, 57.60; H, 4.41; N, 14.60; Found: C, 57.62; H, 4.44; N, 14.65. 

##### *N*-(2-(4-(dimethylamino)phenyl)-4-oxothiazolidin-3-yl)-4-(1*H*-indol-3-yl)-6-methyl-2-thioxo-1,2,3,4-tetrahydropyrimidine-5-carboxamide (**4e**)

Light yellow solid, yield 55%, m.p.170–172 °C. IR (KBr) v max (cm^−1^): 3275 (NH), 1665 and 1695 (2 C=O), 3080 (CH-Ar), 2920 (CH-sp^3^). ^1^H-NMR (300 MHz, DMSO-*d*_6_) 2.3 (s, 3H, CH_3_), 2.5 (s, 3H, 2CH_3_), 3.8 (2H, s, CH_2_ of thiazolidinone ring), 5.9 (1H, s, CH of thiazolidinone ring), 5.1 (s, 1H, pyrimidine), 6.8–8.4 (m, 9H, Ar-H), 9.0, 9.9, 10.2, 10.7 (4 s, 4H, 4NH, D_2_O exchangeable); ^13^C-NMR (300 MHz, DMSO-*d*_6_) 18.2 (CH_3_), 43.6 (N-CH_3_), 58.0 (CH), 37.0 (CH_2_), 55.2 (C-4 pyrimidine), 113.0-140.0 (aromatic Cs), 106.0 (C-5 pyrimidine), 152.2 (C-6 pyrimidine), 165.0, 168.0 (2 C=O), 179.3 (C=S). MS (EI): *m*/*z* 506 [M^+^] (14%). Anal. Calcd. for C_25_H_26_N_6_O_2_S_2_ (506.64): C, 59.27; H, 5.17; N, 16.59; Found: C, 59.28; H, 5.19; N, 16.58.

##### *N*-(2-(4-chlorophenyl)-4-oxothiazolidin-3-yl)-4-(1*H*-indol-3-yl)-6-methyl-2-thioxo-1,2,3,4-tetrahydropyrimidine-5-carboxamide (**4f**)

Yellow solid, yield 65%, m.p. 140–142 °C. IR (KBr) v max (cm^−1^): 3265 (NH), 1660 and 1694 (2 C=O), 3055 (CH-Ar), 2925 (CH-sp^3^). ^1^H-NMR (300 MHz, DMSO-*d*_6_) 2.5 (s, 3H, CH_3_), 3.9 (2H, s, CH_2_ of thiazolodinone ring), 5.9 (1H, s, CH of thiazolidinone ring), 5.1(s, 1H, pyrimidine), 7.1–7.5 (m, 9H, Ar-H), 9.6, 9.9, 10.3, 11.2 (s, 4H, 4NH, D_2_O exchangeable); ^13^C-NMR (300 MHz, DMSO-*d*_6_) 18.2 (CH_3_), 57.3 (CH), 38.3 (CH_2_), 55.2 (C-4 pyrimidine), 111.0-142.0 (aromatic Cs), 106.0 (C-5 pyrimidine), 152.4 (C-6 pyrimidine), 165.1 and 168.0 (2 C=O), 179.5 (C=S); MS(EI): *m*/*z* 497 [M^+^](10 %), 499 [M^+2^] (3%). Anal. Calcd. for C_23_H_20_ClN_5_O_2_S_2_ (498.02): C, 55.47; H, 4.05; N, 14.06; Found: C, 55.44; H, 4.02; N, 14.04.

##### *N*-(2-(3,4-dichlorophenyl)-4-oxothiazolidin-3-yl)-4-(1*H*-indol-3-yl)-6-methyl-2-thioxo-1,2,3,4-tetrahydropyrimidine-5-carboxamide (**4g**)

Yellow crystals, yield 73%, m.p. 150–152 °C. IR (KBr) v max (cm^−1^): 3270 (NH), 1660 and 1698 (2 C=O), 3065 (CH-Ar), 2945 (CH-sp^3^); ^1^H-NMR (300 MHz, DMSO-*d*_6_) 2.6 (s, 3H, CH_3_), 3.95 (s, CH_2_ of thiazolidinone ring), 5.92 (1H, s, CH of thiazolidinone ring), 5.0 (s, 1H, pyrimidine), 6.78–8.27 (m, 8H, Ar-H), 10.0, 10.4, 10.6, 11.0 (4 s, 4H, 4NH, D_2_O exchangeable); ^13^C-NMR (300 MHz, DMSO-*d*_6_) 18.3 (CH_3_), 57.5 (CH), 38.0 (CH_2_). 55.3 (C-4 pyrimidine), 113.0–140.0 (aromatic Cs), 106.0 (C-5 pyrimidine), 152.5 (C-6pyrimidine), 165.9 and 168.0 (2 C=O), 179.5 (C=S). MS (EI): *m*/*z* 531 [M^+^] (16%), 533 [M^+2^] (5%). Anal. Calcd. for C_23_H_19_Cl_2_N_5_O_2_S_2_ (532.46): C, 51.88; H, 3.60; N,13.15; Found: C, 51.85; H, 3.63; N, 13.17.

##### *N*-(2-(4-fluorophenyl)-4-oxothiazolidin-3-yl)-4-(1*H*-indol-3-yl)-6-methyl-2-thioxo-1,2,3,4-tetrahydropyrimidine-5-carboxamide (**4h**)

White solid, yield 75%, m.p.155–158 °C. IR (KBr) v max (cm^−1^): 3280 (NH), 1665 and 1697 (2 C=O), 3075 (CH-Ar), 2955 (CH-sp^3^); ^1^H-NMR (300 MHz, DMSO-*d*_6_) 2.6 (s, 3H, CH_3_), 3.90 (2H, s, CH_2_ of thiazolidinone ring), 5.90 (1H, s, CH of thiazolidinone ring), 5.1 (s, 1H, pyrimidine), 6.6–8.5 (m, 8H, Ar-H), 10.0, 10.2, 10.6, 11.0 (4 s, 4H, 4NH, D_2_O exchangeable); ^13^C-NMR (300 MHz, DMSO-*d*_6_) 18.4 (CH_3_), 57.1 (CH), 38.1 (CH_2_), 55.4 (C-4 pyrimidine), 112.0-143.0 (aromatic Cs), 106.0 (C-5 pyrimidine), 152.5 (C-6 pyrimidine), 165.5 and168.0 (2 C=O), 179.5 (C=S); MS (EI): *m*/*z* 481 [M^+^] (10%). Anal. Calcd. for C_23_H_20_FN_5_O_2_S_2_ (481.56): C, 57.36; H, 4.19; N, 14.54; Found: C, 57.34; H, 4.16; N, 14.52.

### 3.3. Biological Evaluation

#### 3.3.1. In Vitro Cytotoxicity Assay

In vitro cytotoxicity was measured according to the Resazurin Cell Growth Inhibition Assay [53,55,59,60] Alamar Blue or Resazurin (Promega, Mannheim, Germany) reduction assay was used to assess the cytotoxicity of the studied samples. The assay tests cellular viability and mitochondrial function. Briefly, adherent cells were grown in tissue culture flasks, and then harvested by treating the flasks with 0.025% trypsin and 0.25 mM EDTA for 5 min. Once detached, cells were washed, counted, and an aliquot (5 × 10^3^ cells) were placed in each well of a 96-well cell culture plate in a total volume of 100 mL. Cells were allowed to attach overnight and then treated with samples. The final concentration of samples ranged from 0 to 100 mM. After 48 h, 20 mL Resazurin 0.01% *w*/*v* solution was added to each well and the plates were incubated at 37 °C for 1–2 h. Fluorescence was measured on an automated 96-well Infinite M2000 ProTM plate reader (Tecan, Crailsheim, Germany) using an excitation wavelength of 544 nm and an emission wavelength of 590 nm. 5-FU as well as erlotinib were used as standard treatment. Each assay was performed as triplicates. It should be noted that DMSO was used as a solvent control such that the vehicle concentration does not exceed 0.05% in all concentrations used. Cancer cells were treated with gradually increasing concentrations of our compounds from (0.01 μM to 100 μM). The viability was compared based on a comparison with untreated cells. IC_50_ (on cancer cells) were the concentration of sample required to inhibit 50% of the cell proliferation and were calculated from a calibration curve by a linear regression using Microsoft Excel.

#### 3.3.2. In Vivo Antitumor Assay 

In vivo antitumor evaluation was carried out as previously reported [56,60] (see Supplementary Materials).

#### 3.3.3. In Vitro EGFR Inhibitory Assay

The most potent analogs **3g**, **4f**, **4g,** and **4h** against MCF-7, HepG2 and HCT-116 cell lines were further tested for EGFR inhibitory activity using Enzy Chrom TM Kinase Assay Kit (EKIN-400) as previously described [60]. In addition, 20 µL reaction mixture containing the EGFR kinase, ATP, and substrate in the provided assay buffer. Set up a blank control that contains ATP and substrate but no enzyme. Incubate at desired temperature for 30 min. Prepare 900 µL 10µM (adenosine diphosphate) ADP premix by mixing 3 µL of 3 mM standard and 897 µL distilled water. Transfer 20 µL standards into separate wells of the plate. Prepare enough working reagent for each well. Add 40 µL working reagent to each assay well. Tap plate to mix. Incubate at room temperature for 10 min. Read fluorescence intensity at λ_exc_ = 530nm and λ_em_ = 590 nm, and then calculate kinase activity.

#### 3.3.4. Data Analysis

Results are represented as M ± S.E.M of at least three independent experiments. Data analysis has been done using Graph Pad Prism and Sigma Plot 11 software.

### 3.4. Molecular Modeling Procedure 

The crystal structure of the human EGFR co-crystallized with erlotinib (PDB ID: 1M17) was used for the docking analysis. 3D structures of erlotinib and **4g** were obtained using the Discovery Studio software (Accelrys Inc., San Diego, CA, USA). Auto Dock Tools (The Scripps Research Institute, La Jolla, CA, USA) [61] was used to prepare the ligands and receptor as pdbqt files after removing water, adding polar hydrogen atoms and Gasteiger charges respectively. The docking grid box size used was 40 × 40 × 40 Å^3^, encompassing the entire EGFR binding pocket. An exhaustiveness value of 8 was used while keeping the other parameters with their default values. The best docking pose (most stable) was selected for binding mode comparison with that of erlotinib. Visualization of ligand-protein non-covalent interactionswas performed using Discovery Studio software. The schematic 2D representations of enzyme-ligand complexes were generated using LIGPLOT version 2.2.4 (European Molecular Biology Laboratory, Cambridge, UK).

## 4. Conclusions

This study reveals the discovery of novel indolyl pyrimidine hybrids having hydrazide compound **2**, hydrazone compounds **3a**–**h** or condensed thiazolidinone moiety compounds **4a**–**h** with promising antitumor activities. Results of the in vitro cytotoxic evaluation suggest that the hybridization of indole, pyrimidine, and thiazolidinone rings is a promising antitumor scaffold. The most potent antiproliferative compound in this study was compound **4g** in vitro against MCF-7, HepG2 and HCT-116 with IC_50_ = 5.1 ± 1.14, 5.02 ± 1.19 and 6.6 ± 1.40 μM. The presence of electron withdrawal group such as Cl atoms was found to be a potential reason for such activity. Compounds **3g**, **4f, 4g**, and **4h** were the most promising antiproliferative agents against all the tested cell lines. The cytotoxicity of all compounds was more specific to cancer cells compared to WI38 normal cells. Furthermore, compound **4g** showed the highest in vivo antitumor efficacy against EAC tumor bearing mice. In addition, compound **4g** showed a similar effect in in vitro EGFR inhibition assay comparable to erlotinib. Molecular docking of compound **4g** into the binding site of EGFR kinase enzyme was performed to explain the efficacy against EGFR. It was revealed from the docking study that compound **4g** tends to bind more favorably in the EGFR active site than erlotinib. To sum up, the new scaffold reported in this study may represent a new scaffold for developing more effective anticancer agents.

## Figures and Tables

**Table 1:** Cytotoxic activity (IC_50_) of compounds **1**–**4** against MCF-7, HCT-116, HepG2 and WI-38 cell lines.

Compounds	* Cytotoxic Activity IC_50_^a^ (μM)			
	MCF-7	HCT-116	HepG2	WI-38
**1**	26.8 ± 2.20	27.2 ± 1.11	28.8 ± 2.25	30.0 ± 1.20
**2**	21.6 ± 1.8	22.2 ± 1.5	25.5 ± 2.17	40.50 ± 2.25
**3a**	17.0 ± 1.80	15.2 ± 2.32	19.2 ± 1.50	38.23 ± 2.10
**3b**	18.50 ± 2.11	15.6 ± 3.15	19.4 ± 2.19	39.13 ± 1.30
**3c**	18.9 ± 2.00	16.3 ± 4.16	19.9 ± 2.06	37.42 ± 1.49
**3d**	19.2± 2.35	16.6 ± 1.27	20.2 ± 2.33	35.26 ± 2.42
**3e**	19.8 ± 2.70	16.2 ± 1.15	20.4 ± 2.30	40.01 ± 2.31
**3f**	10.7 ± 2.30	10.20 ± 1.18	10.6 ± 2.32	29.82 ± 1.51
**3g**	9.0 ± 2.40	8.5 ± 2.30	10.0 ± 2.46	30.01 ± 3.72
**3h**	10.2 ± 2.01	10.11 ± 2.45	10.4 ± 2.08	28.10 ± 4.22
**4a**	11.2 ± 4.31	12.3 ± 2.17	17.0 ± 4.30	38.03 ± 5.51
**4b**	11.6 ± 3.22	12.6 ± 1.05	18.0 ± 3.20	39.8 ± 4.51
**4c**	12.3 ± 4.08	13.2 ± 1.03	18.5 ± 4.38	29.5 ± 4.02
**4d**	12.6 ± 1.70	13.6 ± 1.09	18.7 ± 1.20	29.9 ± 3.63
**4e**	14.2 ± 0.74	13.7 ± 0.55	18.62 ± 0.94	20.24 ± 5.52
**4f**	8.01 ± 1.83	8.02 ± 0.26	8.9 ± 1.73	19.33 ± 5.87
**4g**	5.1 ± 1.14	5.02± 1.19	6.6 ± 1.40	16.32 ± 3.21
**4h**	6.6 ± 1.28	7.02 ± 0.46	7.5 ± 1.29	18.12 ± 2.06
**5-FU**	5.38 ± 0.24	7.88 ± 0.2	5.34 ± 0.4	5.70 ± 1.50
**erlotinib**	6.65 ± 0.82	7.49 ± 0.65	nd	22.50 ± 0.65

* Three independent experiments were performed for each concentration. ^a^ IC_50_ values represent mean ± SD of three experiments.

**Table 2:** Effect of treatment by compounds **3g**, **4f**, **4g**, and **4h** on MST, % ILS, tumor volume, and viable tumor cell count in EAC tumor bearing mice.

Group	MST (day) ^a^	% ILS ^a^	Tumor Volume (mL) ^a^	Viable Tumor Cell Count (10^6^/mL) ^a^
Normal	nd	nd	nd	Nd
EAC only	16.5	nd	8.01	80.25
**3g**	30	110.0	2.97	48.00
**4f**	37	130.0	2.50	40.70
**4g**	46	230.0	0.89	21.70
**4h**	40	180.0	1.18	28.50
**5-FU**	53.0	265.5	0.80	19.07

^a^ Results are the mean of two experiments. nd, not determined.

**Table 3:** EGFR % inhibition at 10 µM concentration and IC_50_ (µM) of compounds **3g**, **4f**, **4g**, and **4h.**

Compound	% Inhibition of EGFR	IC_50_ (µM) ^a^
**3g**	53	0.50 ± 0.04
**4f**	71	0.38 ± 0.02
**4g**	79	0.25 ± 0.01
**4h**	70	0.39 ± 0.02
**erlotinib**	81	0.30 ± 0.01

^a^ mean of two independent replicates ± SD.

## Data Availability

Not applicable.

## Supplementary Materials

Supplementary data includes molecular modeling, biological evaluation details as well as representative spectral data.

## Abbreviations

MCF-7	Michigan Cancer Foundation-7 cell line
HepG2	Hepatoma G2 cell line
HCT116	human colorectal carcinoma cell line
5-FU	5-Fluorouracil
EGFR	Epidermal Growth Factor Receptor
SAR	Structure-Activity Relationship
HCl	Hydrochloric acid
MS	Mass Spectrometry
IR	Infrared Spectroscopy
NMR	Nuclear Magnetic Resonance
IC_50_	Concentrations of tested compounds that give about 50% inhibition of cell viability
EAC	Ehrlich ascites carcinoma
