# Supplementary material for: Design, Synthesis, Molecular Modeling and Antitumor Evaluation of Novel Indolyl-Pyrimidine Derivatives with EGFR Inhibitory Activity
Source: Molecules. 2021 Mar 25;26(7):1838. doi: 10.3390/molecules26071838 (PMC8037142; doi:10.3390/molecules26071838)
Supplement: Supplementary file 1 [file molecules-26-01838-s001.pdf]

Supplementary material

Design, Synthesis, Molecular Modeling and Antitumor Evaluation of Novel Indolyl-Pyrimidine Derivatives with EGFR Inhibitory Activity

Naglaa Mohamed Ahmed [a,] Mahmoud Moustafa Youns [b,c], Moustafa khames Soltan [d,c] , Ahmed Mohammed Said*[a,e]

[a] Pharmaceutical Organic Chemistry Department, Faculty of Pharmacy, Helwan University, Ein-Helwan, Helwan, Cairo 11795, Egypt

[b] Biochemistry Department, Faculty of Pharmacy, Helwan University, Ein Helwan, Helwan, Cairo 11795, Egypt

[c] Oman college of Health sciences, Muscat, Sultanate of Oman

[d] Medicinal Chemistry Department, Faculty of Pharmacy, Zagazig University, Egypt

[e] Department of Chemistry, University at Buffalo, The State University of New York, Buffalo, NY 14260, USA

*To whom correspondence should be addressed:

Department of Pharmaceutical Organic Chemistry, Faculty of Pharmacy, Helwan University,

Cairo, Egypt E-mail: ahmedchem1982@gmail.com or ahmedmoh@buffalo.edu

1. Docking experiments:

mode	affinity (kcal/mol)	dist from best mode rmsd l.b.	rmsd u.b.
1	-7.3	0.000	0.000
2	-7.3	2.189	4.229
3	-7.3	2.593	4.556
4	-7.1	3.565	6.032
5	-7.0	1.332	2.270
6	-6.9	2.775	5.885
7	-6.9	2.582	6.498
8	-6.8	2.357	4.425
9	-6.8	3.953	8.950

Figure S1. Docking results of erlotinib inside the EGFR binding site

mode	affinity (kcal/mol)	dist from best mode rmsd l.b.	rmsd u.b.
1	-9.6	0.000	0.000
2	-9.2	0.997	1.491
3	-9.2	1.712	4.462
4	-9.1	3.374	6.715
5	-9.1	2.222	4.306
6	-8.9	3.261	7.479
7	-8.8	2.381	3.282
8	-8.8	4.164	6.640
9	-8.8	3.553	7.003

Figure S2. Docking results of compound **4g** inside the EGFR binding site

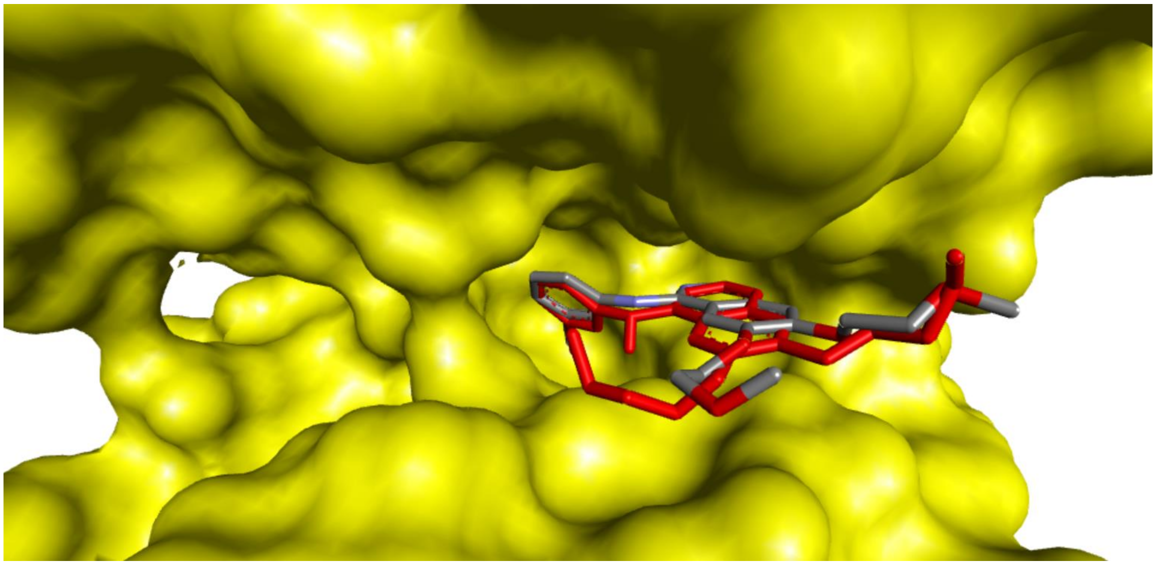

Figure S3. Overlapping the docked structure of erlotinib (red) and the x-ray crystal structure of erlotinib (grey) inside the EGFR binding site

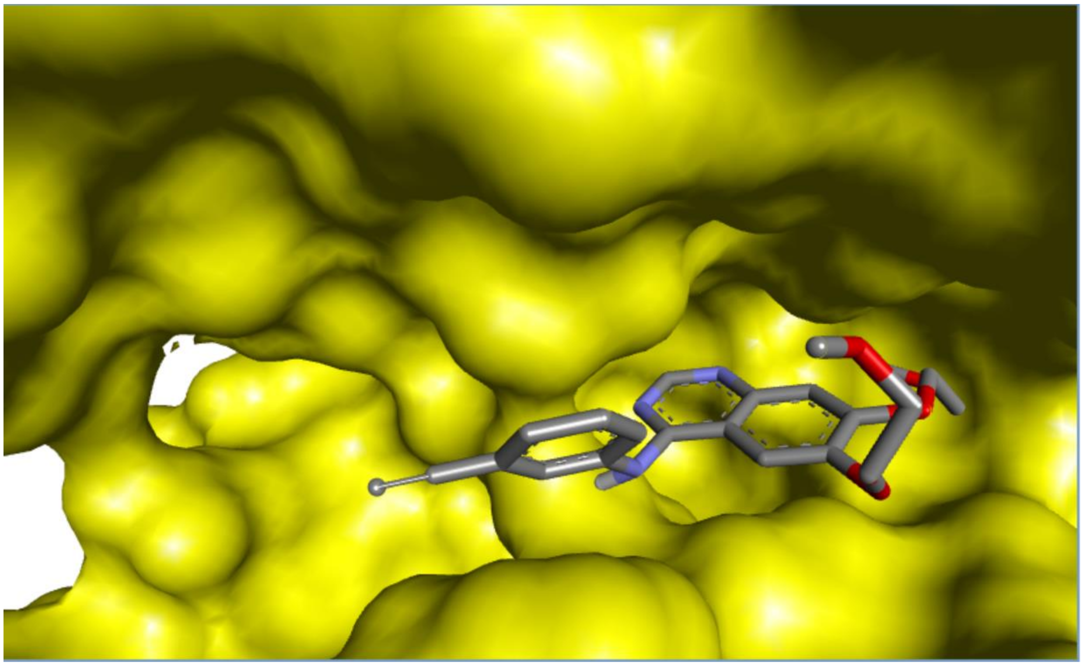

Figure S4. Docked erlotinib inside the EGFR binding site

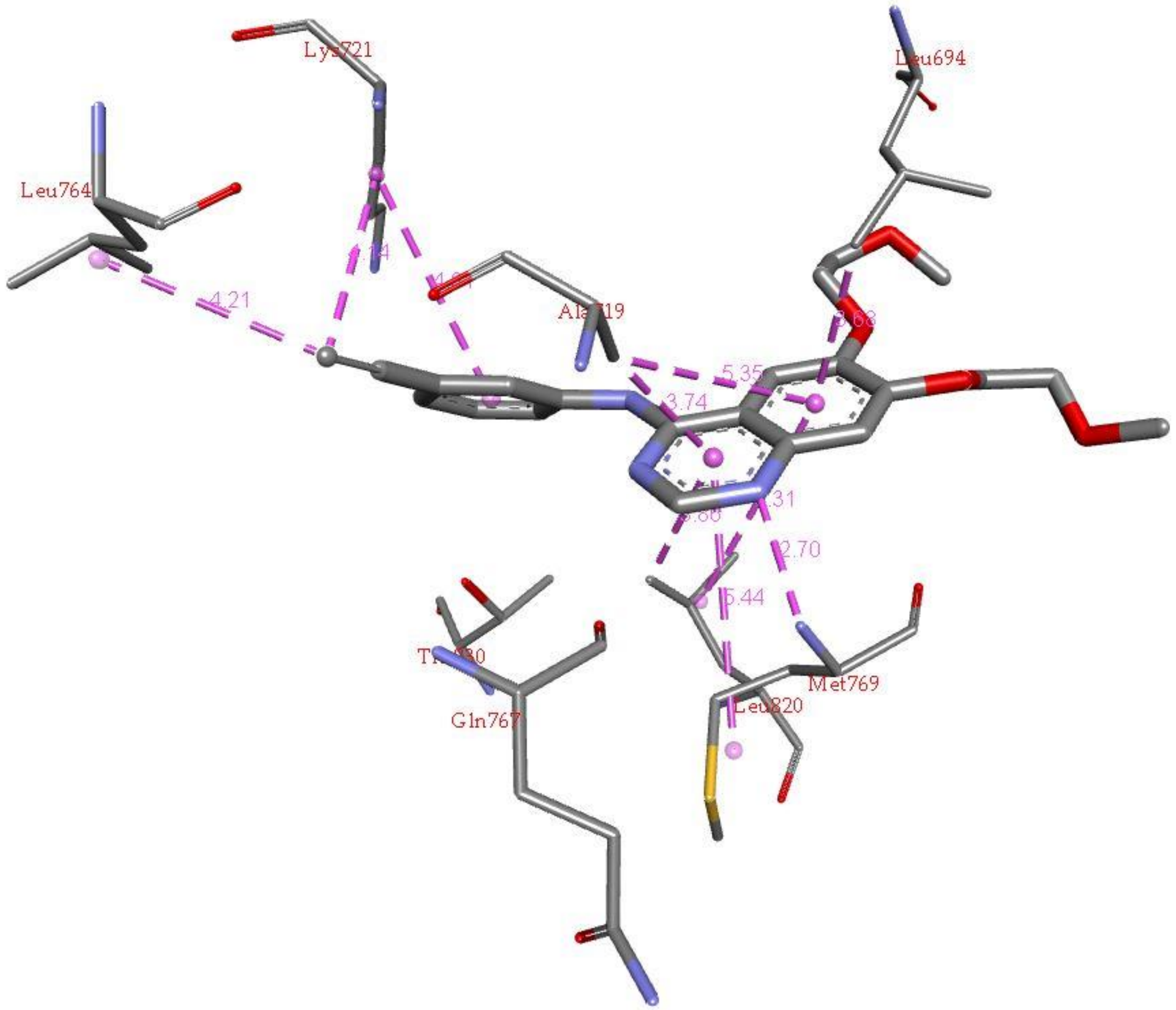

Figure S5. Non-covalent interactions of docked erlotinib inside the EGFR binding site

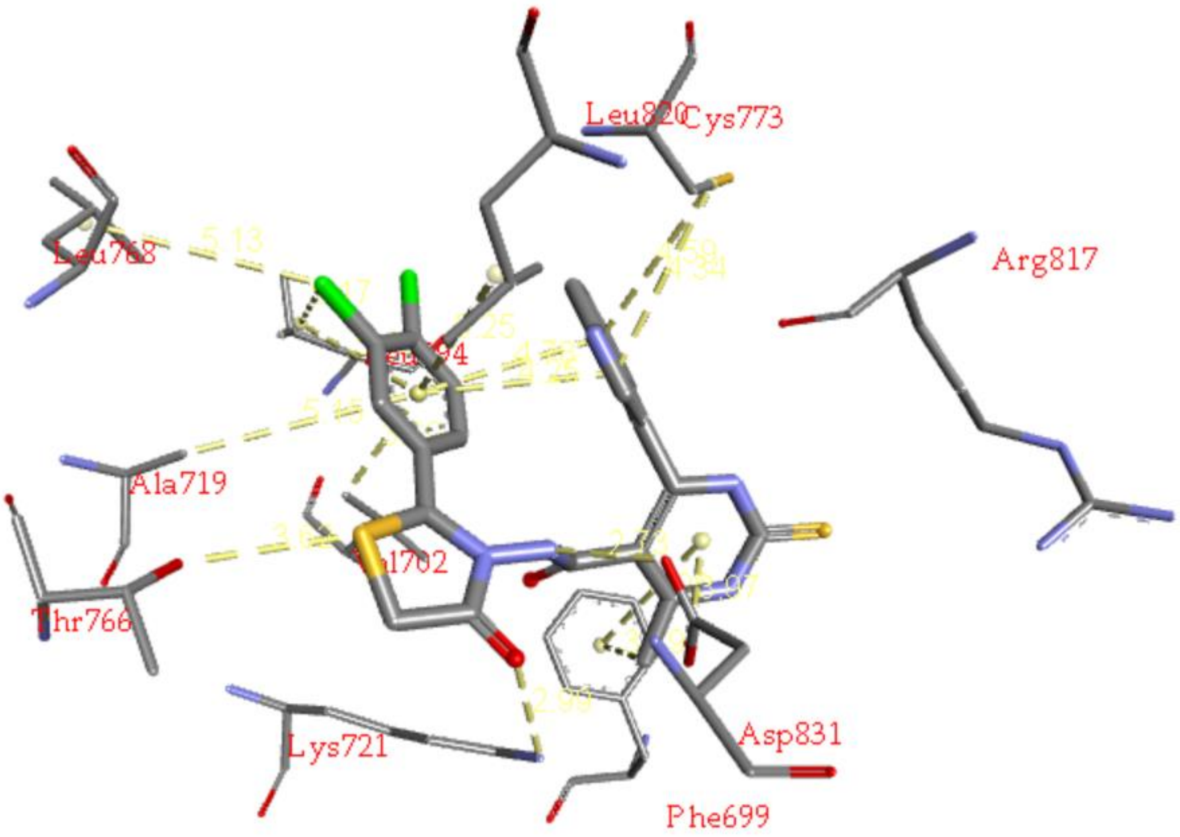

Figure S6. Non-covalent interactions of docked compound **4g** inside the EGFR binding site

2. Biological Evaluation Details:

a) ***In vivo* antitumor assay:** *Materials.* Adult Swiss male mice (weighing 20-25 g) were obtained from the Department of Pharmacology, Faculty of Pharmacy, Mansoura University, Egypt. They were kept in microlon boxes at temperature 25 ± 2 °C with a regular 12 hrs light/dark cycle. In addition, food and water were available. EAC cells (procured from NCI, Cairo, Egypt) were harvested and prepared, then their total number was counted.

Procedure. 7 Groups of mice (*n* = 4) were used.

Group 1: Negative control (no EAC cells) - received normal saline.

Group 2: Positive control (EAC cells) - received normal saline.

Group 3: EAC cells - treated with compound **3g** (0.5 mg/mouse).

Group 4: EAC cells - treated with compound **4f** (0.5 mg/mouse).

Group 5: EAC cells - treated with compound **4g** (0.5 mg/mouse).

Group 6: EAC cells - treated with compound **4h** (0.5 mg/mouse).

Group 7: EAC cells - treated with 5-fluorouracil (0.5 mg/mouse).

2 x 10^6 EAC cells were injected intraperitoneally in each mouse (groups 2-7). After one day, compounds **3g**, **4f**, **4g** and **4h**, and 5-fluorouracil were administered to mice for nine days, then blood samples were withdrawn for assessment of blood count.

Determination of tumor volume

The ascetic fluid was gathered from the peritoneal cavity and its volume was determined. It was then centrifuged, and the packed tumor cell volume was measured.

Determination of viable tumor cell count

100 μL Sample of EAC cells (from three mice per group) was utilized and diluted twenty times with saline. Cells were stained with trypan blue, viable cells are not stained, while the dead ones are stained. The number of viable cells was calculated.

3. Chemistry Details: Representative Spectra.

DMSO-H1

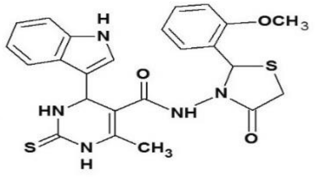

4c

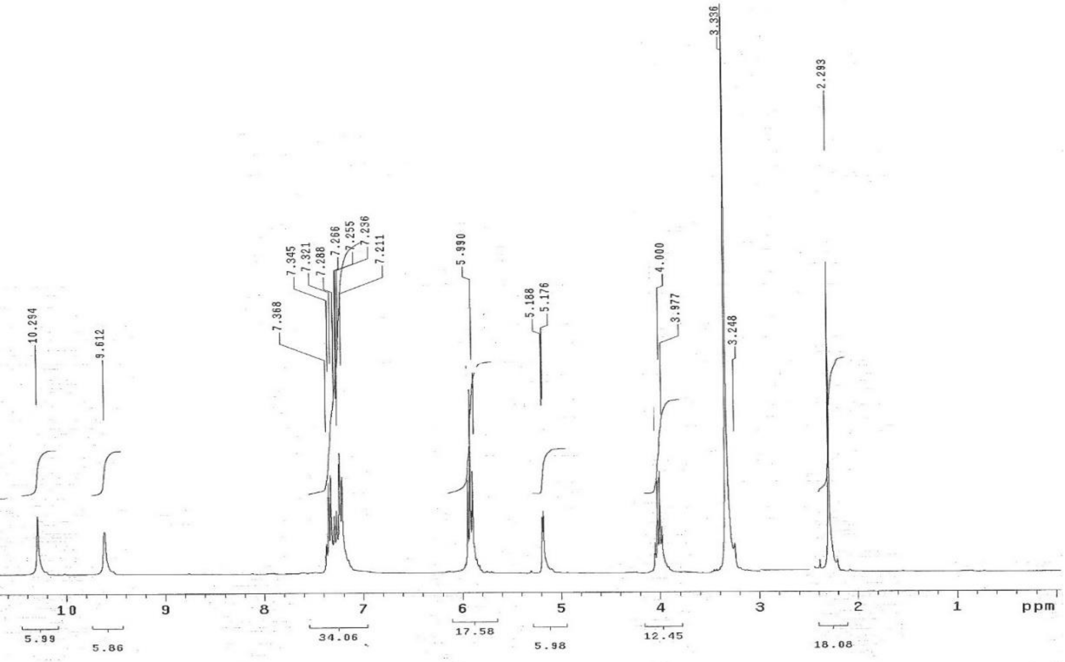

10.294
9.612
7.368
7.345
7.321
7.288
7.266
7.255
7.236
7.211
5.990
5.188
5.176
4.000
3.977
3.336
3.248
2.293
13
12
11
10
9
8
7
6
5
4
3
2
1
ppm
10.92
5.99
5.86
34.06
17.58
5.98
12.45
18.08

-DMSO-H1

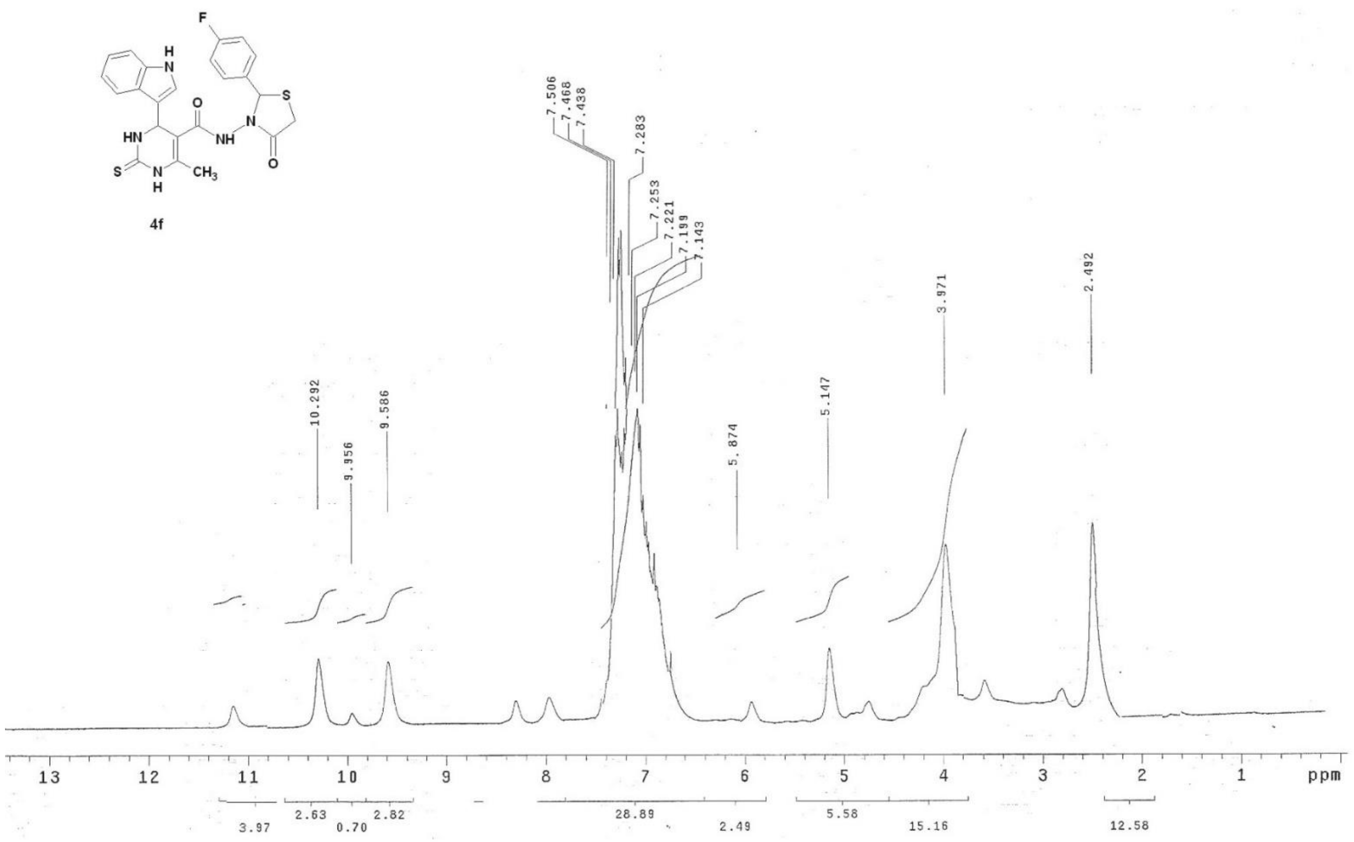

4f
F
H
N
HN
S
H
CH3
O
NH
S
O
7.506
7.468
7.438
7.283
7.253
7.221
7.199
7.143
10.292
9.956
9.586
5.874
5.147
3.971
2.492
13
12
11
10
9
8
7
6
5
4
3
2
1
ppm
3.97
2.63
0.70
2.82
28.89
2.49
5.58
15.16
12.58

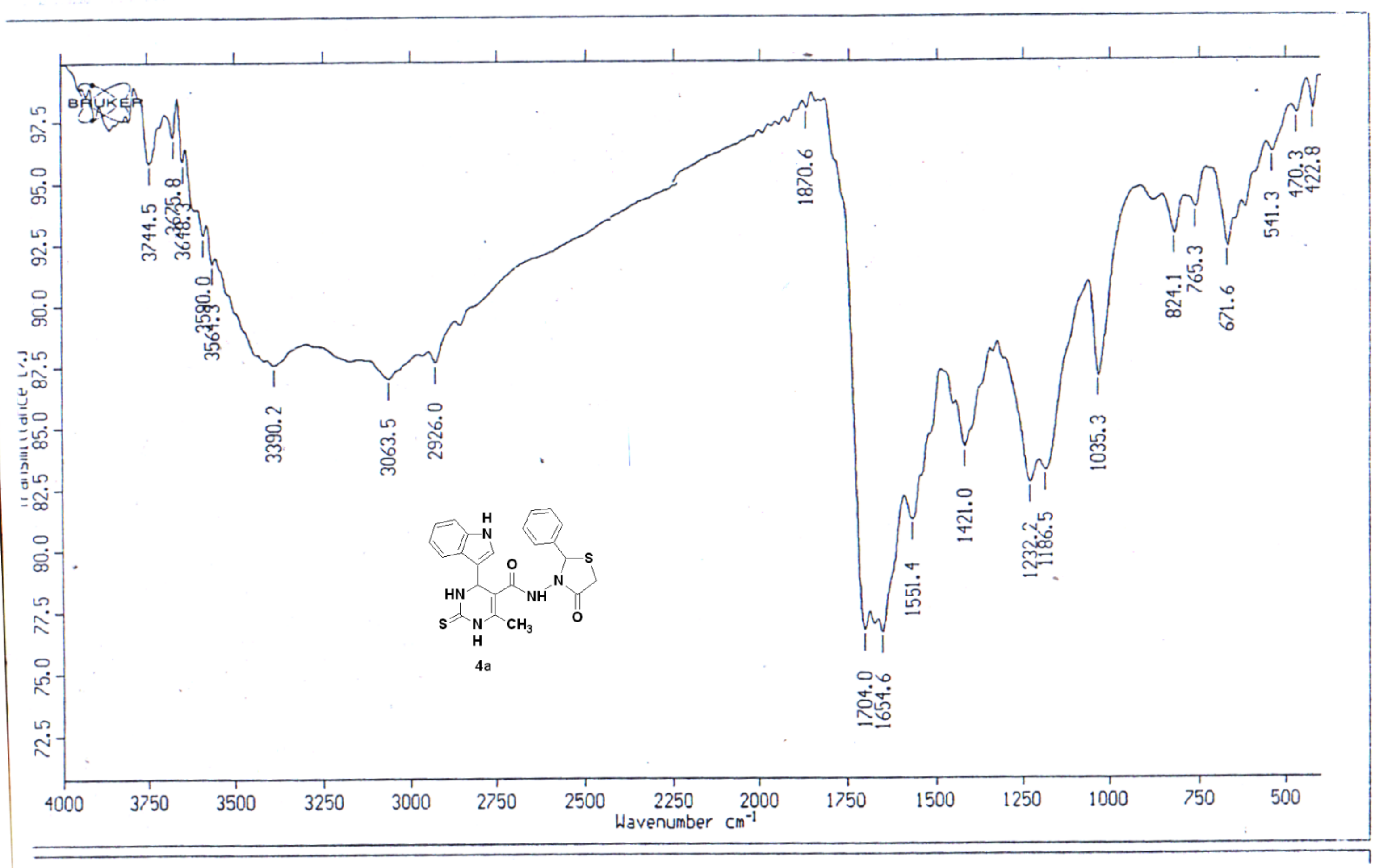

BRUKER
Transmittance [%]
97.5
95.0
92.5
90.0
87.5
85.0
82.5
80.0
77.5
75.0
72.5
4000
3750
3500
3250
3000
2750
2500
2250
2000
1750
1500
1250
1000
750
500
Wavenumber cm-1
3744.5
3675.8
3648.3
3590.0
3561.3
3390.2
3063.5
2926.0
1870.6
1704.0
1654.6
1551.4
1421.0
1232.2
1186.5
1035.3
824.1
765.3
671.6
541.3
470.3
422.8
4a

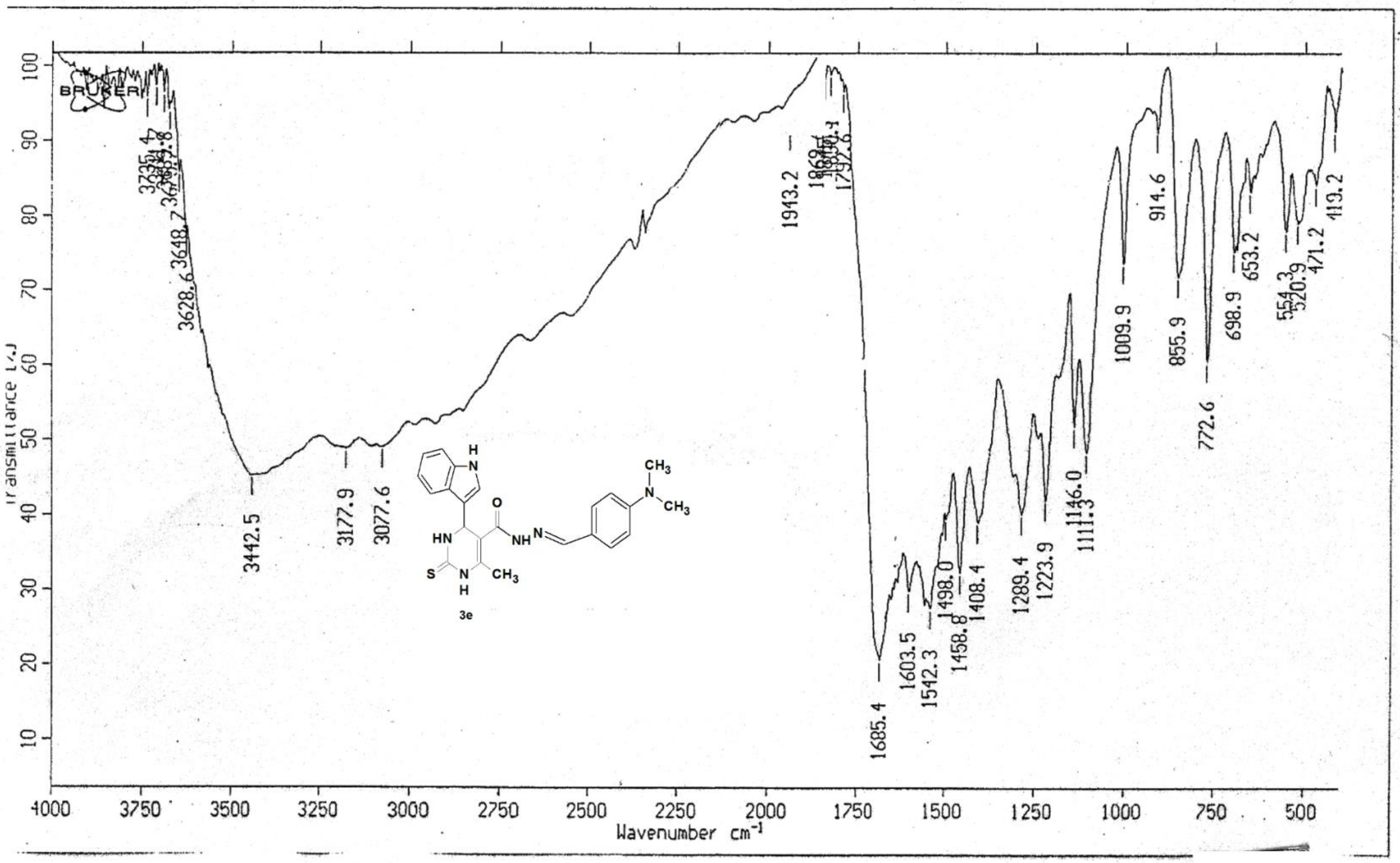

Transmittance [%]
100
90
80
70
60
50
40
30
20
10
4000
3750
3500
3250
3000
2750
2500
2250
2000
1750
1500
1250
1000
750
500
Wavenumber cm-1
3442.5
3177.9
3077.6
3628.6
3648.2
1943.2
1685.4
1603.5
1542.3
1498.0
1458.8
1408.4
1289.4
1223.9
1146.0
1111.3
1009.9
914.6
855.9
772.6
698.9
653.2
554.3
520.9
471.2
419.2
3e

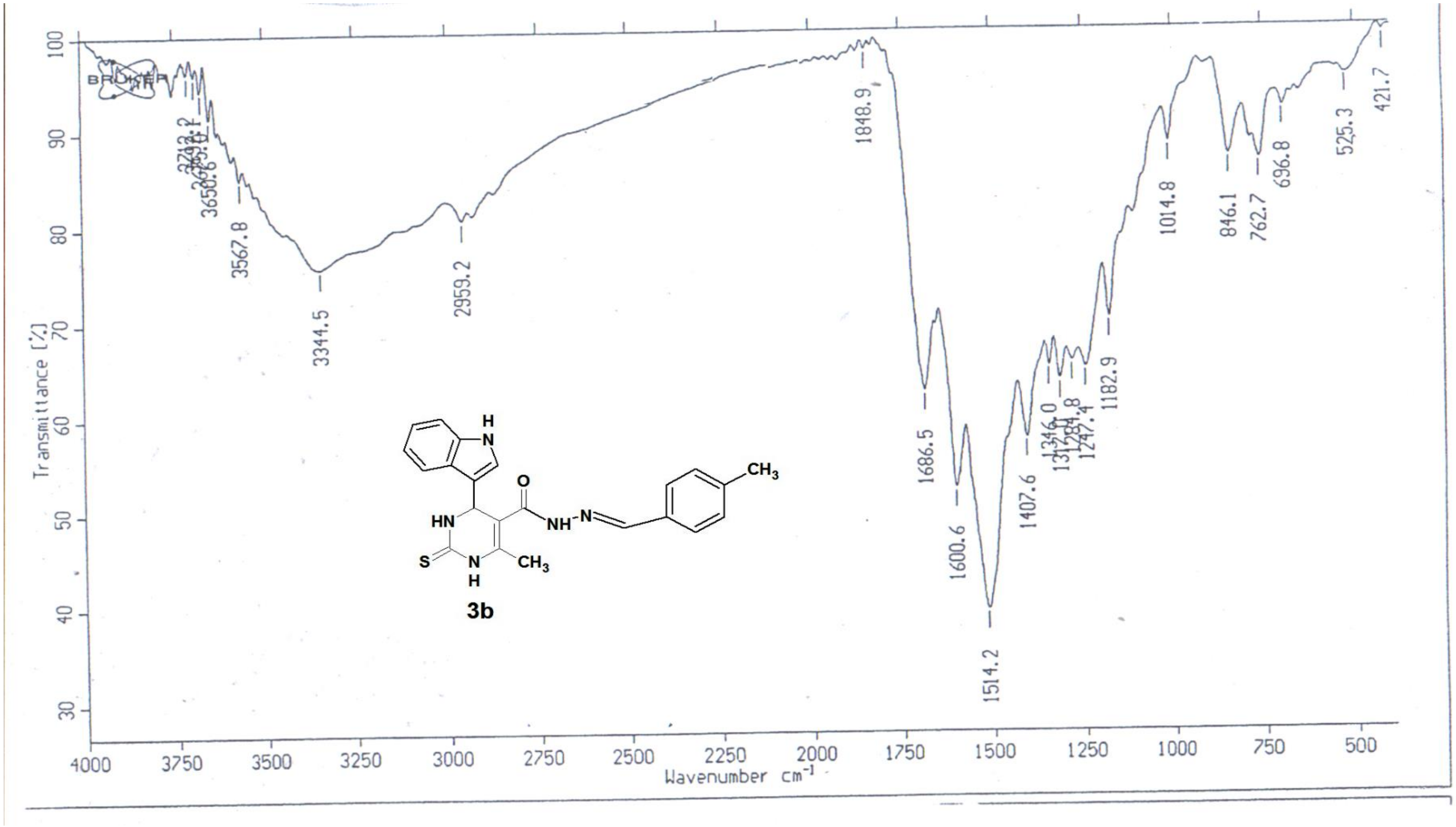
Transmittance [%]
Wavenumber cm-1
3344.5
2959.2
3567.8
1848.9
1686.5
1600.6
1514.2
1407.6
1346.0
1284.8
1247.4
1182.9
1014.8
846.1
762.7
696.8
525.3
421.7
3b

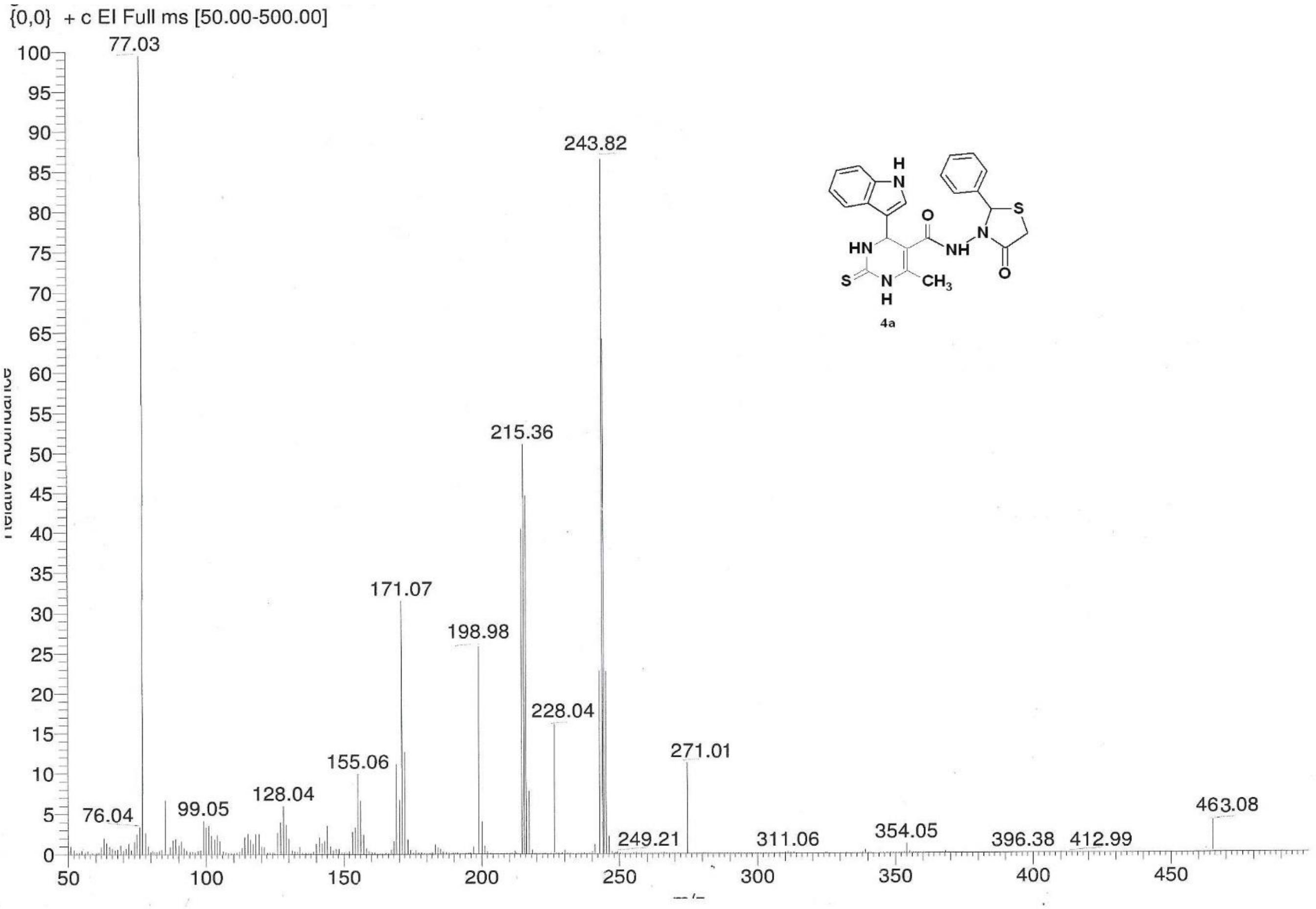
{0,0} + c EI Full ms [50.00-500.00]
Relative Abundance
77.03
76.04
99.05
128.04
155.06
171.07
198.98
215.36
228.04
243.82
249.21
271.01
311.06
354.05
396.38
412.99
463.08
0
5
10
15
20
25
30
35
40
45
50
55
60
65
70
75
80
85
90
95
100
50
100
150
200
250
300
350
400
450
m/z
4a

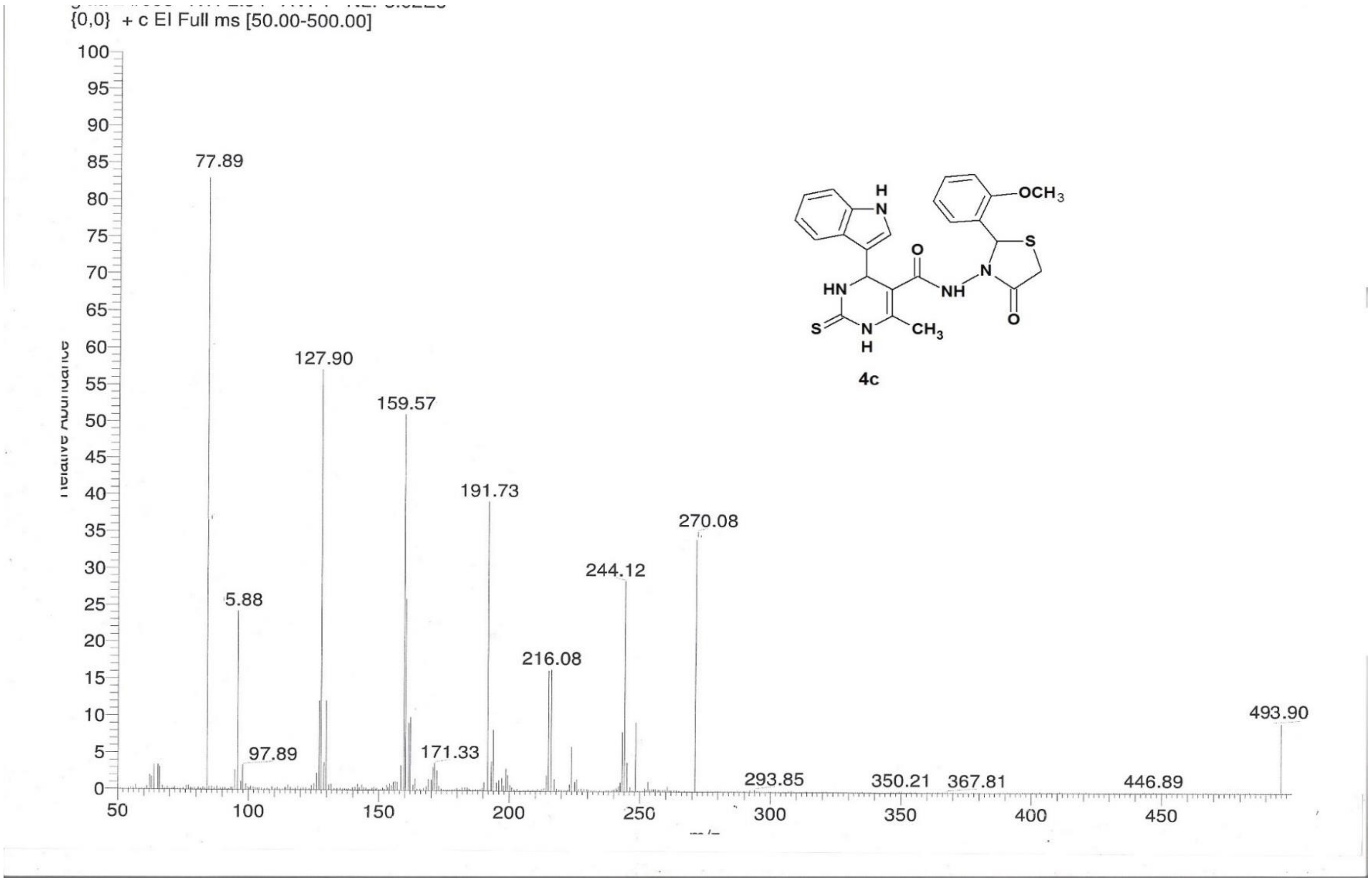
{0,0} + c EI Full ms [50.00-500.00]
Relative Abundance
77.89
5.88
97.89
127.90
159.57
171.33
191.73
216.08
244.12
270.08
293.85
350.21
367.81
446.89
493.90
HN
S
N
H
CH3
O
NH
N
S
O
OCH3
4c

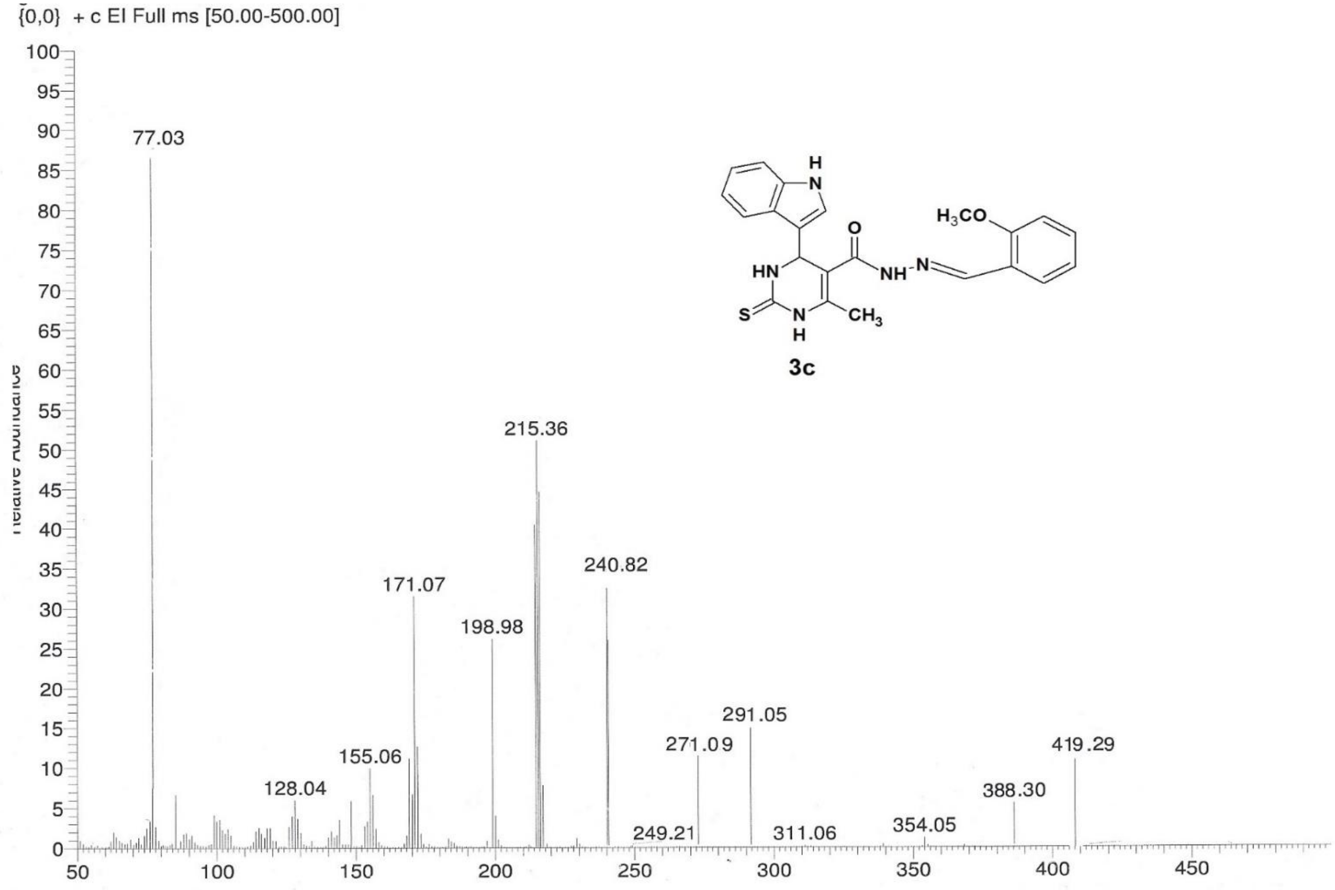
{0,0} + c EI Full ms [50.00-500.00]
Relative Abundance
77.03
128.04
155.06
171.07
198.98
215.36
240.82
249.21
271.09
291.05
311.06
354.05
388.30
419.29
H
N
O
HN
NH
N
H_3CO
S
N
H
CH_3
3c
